# Large Language Models as Clinical Nutrition Decision Tools: Quantitative Bias and Guideline Deviation in Type 2 Diabetes Meal Planning

**DOI:** 10.3390/healthcare14060739

**Published:** 2026-03-13

**Authors:** Pinar Ece Karakas, Aysenur Calik, Ayse Betul Bilen, Kardelen Kandemir, Muveddet Emel Alphan

**Affiliations:** 1 Faculty of Health Sciences, Department of Nutrition and Dietetics, Istanbul Atlas University, Istanbul 34403, Türkiye; ayse.demirbas@atlas.edu.tr (A.B.B.); kardelen.yoldas@atlas.edu.tr (K.K.); emel.alphan@atlas.edu.tr (M.E.A.); 2 PhD Program in Nutrition and Dietetics, Institute of Graduate Studies, Istanbul Halic University, Istanbul 34060, Türkiye

**Keywords:** artificial intelligence, large language models, type 2 diabetes, medical nutrition therapy, dietary planning, guideline adherence

## Abstract

**Highlights:**

**What are the main findings?**

- Large language models generated dietary plans for type 2 diabetes that differed substantially from a guideline-based, dietitian-designed reference diet, particularly in energy intake and dietary fiber adequacy.
- Most AI-generated diets followed a low-energy, lower-carbohydrate, higher-protein, and insufficient-fiber pattern, with limited evidence of individualized medical nutrition therapy.

**What are the implications of the main findings?**

- AI-generated dietary plans for diabetes management should not replace professional medical nutrition therapy without expert evaluation.
- Careful clinical validation and guideline-based refinement are required before integrating large language models into routine diabetes care.

**Abstract:**

**Background/Objectives:** Large language models (LLMs) are increasingly used as decision support tools in clinical nutrition, including meal planning for individuals with type 2 diabetes mellitus (T2DM). However, the clinical safety, quantitative accuracy, and guideline adherence of AI-generated dietary plans remain uncertain. This study aimed to evaluate systematic bias and agreement between LLM-generated diets and a guideline-concordant reference diet, and to assess whether current LLMs can function as reliable clinical nutrition decision support tools in T2DM. **Methods:** Six widely used LLMs generated standardized three-day, 1800 kcal dietary plans for T2DM using an identical prompt. Each day was treated as an independent observation (*n* = 18). Energy and macronutrient contents were analyzed using professional nutrition software and compared with a dietitian-designed reference diet based on ADA, EASD, IDF, and national guidelines. Agreement was evaluated using Bland–Altman analysis, proportional bias assessment, and intraclass correlation coefficients. Guideline adherence and clinical appropriateness were independently scored by registered dietitians. **Results:** Most LLM-generated diets systematically deviated from the reference diet, with lower total energy, reduced carbohydrate and fiber content, and variable protein distribution. Bland–Altman analyses demonstrated significant bias and wide limits of agreement for key nutrients, indicating clinically meaningful discrepancies. Guideline adherence scores varied substantially across models, with only one model showing relatively consistent performance. Inter-rater reliability between dietitians was high (ICC = 0.806). **Conclusions:** Current LLMs exhibit systematic quantitative bias and inconsistent guideline adherence when used for T2DM meal planning. AI-generated dietary plans are not interchangeable with dietitian-guided medical nutrition therapy and may pose clinical risks if used without professional oversight. Careful validation, domain-specific fine-tuning, and integration within supervised clinical workflows are required before implementation in diabetes care.

## 1. Introduction

Diabetes is a chronic metabolic disease affecting more than half a billion people worldwide and is associated with a rapidly increasing prevalence, as well as substantial morbidity, mortality, and healthcare expenditures. The global rise in physical inactivity and sedentary lifestyles, the widespread adoption of energy-dense and nutrient-poor dietary patterns commonly referred to as the Western diet, and the growing aging population are among the primary drivers of the increasing incidence of type 2 diabetes mellitus (T2DM) [1]. Medical nutrition therapy (MNT) is a cornerstone of T2DM management and plays a critical role in achieving glycemic control, reducing cardiometabolic risk factors, and improving quality of life [2]. International diabetes guidelines emphasize that MNT for T2DM should focus on regulating both the amount and quality of carbohydrate intake, increasing dietary fiber consumption, ensuring an appropriate distribution of healthy fatty acids, and maintaining energy balance, while also highlighting the importance of individualized and sustainable nutrition therapy [3]. However, factors such as time constraints, limited access to dietitians, patient preferences, and educational level may hinder the translation of evidence-based recommendations into individualized, sustainable, and feasible dietary plans in clinical practice, thereby reducing the effectiveness of MNT [4]. Against this background, the growing need for accessible, timely, and individualized nutrition support has prompted increasing interest in scalable digital tools, including large language models (LLMs), as potential aids for meal planning and dietary guidance [5,6].

In recent years, LLMs have gained increasing attention in healthcare, including nutrition, because of their ability to generate rapid, detailed, and user-responsive recommendations. In nutrition practice, these tools have attracted considerable interest from both healthcare professionals and the public, particularly because of their potential to generate meal plans, summarize dietary guidance, and provide apparently personalized recommendations based on user prompts [5,6]. However, important concerns remain regarding the reliability of artificial intelligence (AI)-generated dietary advice. Across health-related applications, LLMs have been reported to produce quantitatively inaccurate outputs, hallucinated content, inconsistent recommendations, and responses that may drift from evidence-based clinical guidelines. In chronic conditions such as T2DM, where dietary management requires sustained accuracy, balance, and individualization, such limitations may reduce clinical usefulness and potentially introduce patient safety risks [7,8,9].

In the literature, studies that systematically evaluate dietary plans generated by different LLMs in accordance with the principles of MNT for T2DM are limited. In particular, relatively few studies have examined whether such plans are quantitatively aligned with evidence-based nutritional targets and clinically applicable when compared with an expert dietitian-designed reference diet. Therefore, the aim of this study was to evaluate dietary plans generated by different LLMs within the framework of MNT for T2DM in terms of nutrient adequacy, compliance with current dietary guidelines, and clinical applicability, and to compare the obtained outputs with a reference diet plan prepared by an expert dietitian based on up-to-date, evidence-based guidelines. By combining nutrient-level assessment with expert evaluation, this study aims to contribute to ongoing discussions on the safe, evidence-based, and clinically responsible use of AI-based tools in nutrition and diabetes management.

## 2. Materials and Methods

### 2.1. Study Design

**Figure 1:**
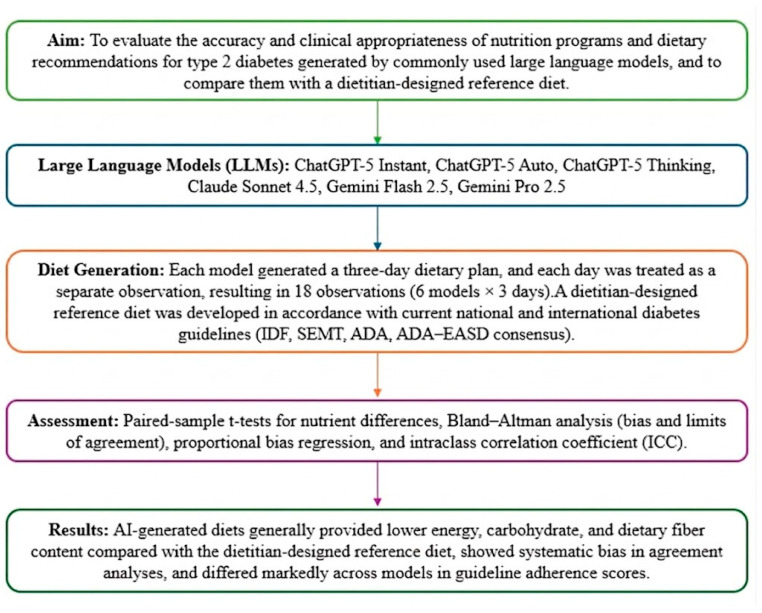
Schematic representation of the study design and analysis process. IDF: International Diabetes Federation; SEMT: Society of Endocrinology and Metabolism of Turkey; ADA: American Diabetes Association; ADA-EASD: American Diabetes Association–European Association for the Study of Diabetes.

This study was designed as a quantitative agreement and bias analysis to evaluate the clinical safety and guideline concordance of LLMs when used as nutrition decision support tools in T2DM. Three-day dietary plans generated by widely used LLMs were compared with a dietitian-designed, guideline-concordant reference diet. Nutrient composition was quantitatively analyzed, and agreement was assessed to determine clinical interchangeability. In addition, the adherence of AI-generated dietary recommendations to national and international diabetes nutrition guidelines was independently evaluated by registered dietitians using a structured scoring system. Details of the study design and analytical workflow are presented in Figure 1 and the following subsections.

### 2.2. Generation of AI-Based Dietary Plans

For this study, six versions of three widely used LLMs were selected: ChatGPT-5 Instant [10], ChatGPT-5 Auto [11], ChatGPT-5 Thinking [12], Claude Sonnet 4.5 [13], Gemini Flash 2.5 [14], and Gemini Pro 2.5 [15]. The selected models were chosen to represent different levels of accessibility (free and paid), distinct inference modes (rapid response, automatic, and advanced reasoning), and multiple developer platforms. This approach was intended to enable a more comprehensive and representative evaluation of LLM outputs under real-world usage conditions. To further minimize the influence of prior user interactions, a new and dedicated account was created for each platform, ensuring that model responses were not influenced by previous conversation history or personalized learning. Model-specific parameters such as temperature settings or system-level instructions were not manually adjusted, and all models were used under their default interface configurations as available to general users at the time of data collection.

All models were instructed using the following prompt (translated from Turkish): “Prepare a 3-day 1800 kcal diet plan for individuals with type 2 diabetes based on national and international nutrition guidelines. Also include the macronutrient and micronutrient values, their proportions, and energy contributions. In addition, provide detailed nutrition recommendations for type 2 diabetes.”

Each model generated a three-day dietary plan. Each day was treated as a separate observation, resulting in a total of 18 observations (6 models × 3 days) (Text S2: Full Version of Reference Diet and AI-Generated Diets).

A single standardized clinical scenario (fixed energy target and disease profile) was intentionally used to isolate intrinsic model performance and eliminate variability arising from patient heterogeneity. This approach allowed for a controlled assessment of systematic bias and agreement across models under identical clinical conditions.

All model interactions were conducted as single-turn queries in newly initiated independent sessions. No follow-up prompts, clarification requests, retries, or iterative refinements were allowed, and the first complete output from each model was retained for analysis. Incomplete or non-interpretable responses would have been excluded and documented as protocol deviations; no such cases were encountered in this study. No user-defined system prompts were used. Model parameters such as temperature or other generation settings were not visible or user-configurable for all platforms and therefore could not be standardized. Before BeBiS [16] analysis, AI-generated diet plans were converted into a standardized food-record format. Food items and portion descriptions were entered as closely as possible to the model-generated outputs using foods available in the BeBiS [16] database, and no food substitutions were required.

### 2.3. Reference Diet Preparation

The reference diet was designed by a registered dietitian (RD) with clinical experience in diabetes nutrition therapy, following the same specifications provided in the standardized AI prompt (three days, 1800 kcal) (Text S2: Full Version of Reference Diet and AI-Generated Diets). The diet was developed in accordance with current national and international evidence-based diabetes nutrition guidelines, including those issued by the International Diabetes Federation (IDF) [1], the Society of Endocrinology and Metabolism of Turkey (SEMT) [17], the American Diabetes Association (ADA) [3], and the ADA–European Association for the Study of Diabetes (EASD) consensus reports [2]. Although current guidelines do not specify a standard amount for energy and macronutrient intake and emphasize the need for individualization, they recommend that macronutrient distribution should provide 45–60% of total energy from carbohydrates, 10–20% from protein, and 20–35% from fat. In addition, recent guidelines recommend a minimum dietary fiber intake of 14 g per 1000 kcal of energy [3,17]. The reference diet was designed in accordance with current dietary guidelines and structured to include three main meals and three snacks. The diet was characterized by a high dietary fiber content and a predominant inclusion of vegetables, fruits, whole grains, and legumes, while the intake of refined carbohydrates, trans fats, and saturated fats was deliberately limited. The reference diet served as a guideline-concordant benchmark for comparison with the AI-generated dietary plans.

### 2.4. Nutrient Analysis of Dietary Plans

The energy and nutrient contents of the three-day dietary plans generated by the different LLMs were analyzed by the researchers using BeBiS 9.0 software [16]. The analyzed dietary components included total energy, carbohydrate, protein, fat, and dietary fiber. All food items proposed by the AI-generated diets were available in the BeBiS 9.0 [16] database; therefore, no food substitutions were required during the analysis.

Each day of the AI-generated dietary plans was treated as a separate dietary record, resulting in a total of 18 AI-generated diets. These individual daily diets were compared with the reference diet in the agreement analyses. In addition, mean values for energy and nutrient contents were calculated across all 18 AI-generated diets for comparative analyses.

Although the standardized prompt explicitly requested micronutrient values, a systematic qualitative review of model outputs revealed that the majority of LLMs either omitted micronutrient data entirely or reported only partial values for selected nutrients (e.g., sodium, calcium). Due to the inconsistent and incomplete nature of micronutrient reporting across models, a quantitative comparative analysis of micronutrient adequacy was not feasible. This limitation in model output itself is reported as a qualitative finding relevant to clinical safety.

### 2.5. Evaluation of Guideline Adherence and Dietary Recommendations

The content and accuracy of the dietary plans and accompanying dietary recommendations generated by the different large language models were evaluated based on national and international diabetes nutrition guidelines. These guidelines included those published by IDF, SEMT, ADA, and ADA-EASD consensus reports (Table 1).

Guideline adherence was assessed by examining key dietary domains derived from the recommendations, including energy appropriateness, macronutrient distribution and dietary fiber adequacy. The qualitative content of dietary recommendations was independently evaluated by registered dietitians using a structured scoring system, as described in the Statistical Analysis section.

### 2.6. Statistical Analysis

Statistical analyses were performed using IBM SPSS Statistics version 24.0 [18]. Descriptive statistics were calculated for energy and nutrient values derived from the BeBiS 9.0 [16] analyses of the 18 AI-generated dietary records and are presented as mean ± standard deviation.

As each AI-generated daily diet was directly compared with the dietitian-designed reference diet, paired-sample *t*-tests were used to assess differences in mean energy, carbohydrate, protein, fat, and dietary fiber contents. Prior to analysis, the normality of the differences between paired measurements was evaluated. Deviations of AI-generated diets from the reference diet were visually presented using box-and-whisker plots.

Agreement between the reference diet and AI-generated diets for energy, carbohydrate, protein, fat, and dietary fiber was assessed using Bland–Altman analysis, following the method described by Bland and Altman [19]. For each dietary component, the mean difference (bias) and the 95% limits of agreement (LOA) were calculated, and Bland–Altman plots were generated using the differences between each AI-generated daily diet and the reference diet against their means.

The appropriateness of dietary recommendations generated by the different language models in relation to national and international diabetes guidelines was independently evaluated by two registered dietitians with clinical experience in diabetes nutrition therapy. Recommendations were scored on a 10-point scale based on predefined criteria derived from the literature. Inter-rater reliability was assessed using the Intraclass Correlation Coefficient (ICC). A *p*-value < 0.05 was considered statistically significant for all analyses.

To assess potential within-model clustering of daily dietary outputs, sensitivity analyses were performed using linear mixed-effects models with model identity included as a random intercept. In addition, intraclass correlation coefficients (ICCs) were calculated to evaluate similarity among the three daily diets generated by each language model. These analyses were conducted to examine whether daily dietary plans could reasonably be treated as independent observations in the primary analyses. Detailed results are provided in the Supplementary Materials.

To facilitate clinical interpretation of the Bland–Altman results, clinically meaningful thresholds were considered based on established nutritional guidelines. A deviation of ≥10% from the target daily energy intake (equivalent to approximately ±180 kcal for an 1800 kcal diet) was considered clinically relevant. For dietary fiber adequacy was evaluated relative to guideline recommendations of approximately 14 g per 1000 kcal, corresponding to roughly 25–30 g/day for an 1800 kcal diet.

## 3. Results

### 3.1. Energy and Nutrient Content of AI-Generated Diets

The energy and nutrient composition of dietary plans generated by different LLMs under identical standardized conditions were quantitatively compared with a guideline-concordant reference diet designed by a registered dietitian. According to the BeBiS 9.0 analysis, the reference diet provided 48.0% of total energy from carbohydrates, 17.9% from protein, and 33.9% from fat, consistent with current diabetes nutrition recommendations.

Across models, systematic deviations from the target energy prescription were observed. Diets generated by Gemini 2.5 Pro, ChatGPT-5 Auto, and Gemini 2.5 Flash provided significantly lower total energy than the reference diet (*p* < 0.05), indicating underestimation of caloric targets under standardized conditions.

Carbohydrate content was consistently lower in most AI-generated diets compared with the reference diet, with statistically significant differences across all models except Claude Sonnet 4.5 (*p* < 0.05). This pattern reflects a systematic shift toward lower carbohydrate distribution relative to guideline recommendations.

Protein content demonstrated inter-model variability, with ChatGPT-5 Instant and ChatGPT-5 Thinking generating significantly higher protein amounts than the reference diet (*p* < 0.05). No statistically significant differences were detected for total fat content (*p* > 0.05).

Regarding dietary fiber, most LLM-generated diets contained lower fiber levels compared with the reference diet; however, Claude Sonnet 4.5 showed no statistically significant difference (*p* > 0.05). Overall, these findings indicate consistent quantitative discrepancies in key macronutrient targets across models (Table 2 and Table S1).

### 3.2. Deviations from the Reference Diet: Boxplot Analysis

**Figure 2:**
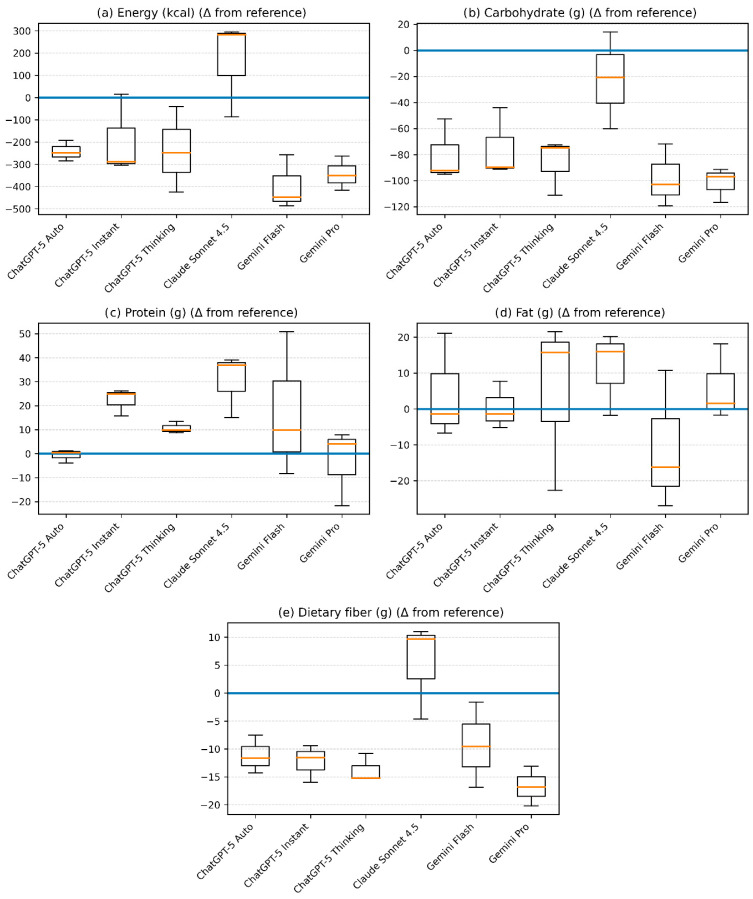
Deviations in energy and nutrient content of AI-generated dietary plans relative to the reference diet. Box-and-whisker plots show differences for (**a**) energy, (**b**) carbohydrate, (**c**) protein, (**d**) fat, and (**e**) dietary fiber across large language models. The horizontal line at zero represents the reference diet; values below and above zero indicate lower and higher intakes, respectively.

Deviations in energy and nutrient contents of the three-day dietary plans generated by different LLM relative to the reference diet are presented using box-and-whisker plots in Figure 2. In terms of energy content, the diets generated by Claude Sonnet 4.5 exhibited the widest range of deviation from the reference diet, with values ranging approximately from −100 to +300 kcal. In contrast, Gemini 2.5 Flash showed a pronounced negative deviation in energy content, with values around −500 kcal. For the remaining models, energy deviations were predominantly distributed between −200 and −400 kcal.

All LLMs demonstrated negative deviations in carbohydrate content compared with the reference diet. The greatest reductions in carbohydrate content were observed in Gemini 2.5 Pro and Gemini 2.5 Flash, with deviations ranging approximately from −90 to −120 g. Protein content showed smaller deviations overall, with diets generated by ChatGPT-5 Auto providing protein amounts closest to the reference diet, typically within a range of −5 to +5 g.

Regarding fat content, Gemini 2.5 Flash exhibited the largest negative deviations, with fat intake approximately 15–25 g lower than the reference diet. In contrast, Claude Sonnet 4.5 showed a consistent positive deviation in fat content, with values approximately 10–20 g higher than the reference diet, while the remaining models displayed fat contents broadly comparable to the reference diet.

With respect to dietary fiber, all LLMs except Claude Sonnet 4.5 generated diets with lower fiber content than the reference diet. Fiber deviations for most models were consistently negative and generally ranged from approximately −10 to −20 g, whereas diets generated by Claude Sonnet 4.5 were centered around the reference value and, in several cases, exhibited modest positive deviations. These distribution patterns are consistent with the mean differences reported in Table 2.

### 3.3. Agreement Between AI-Generated Diets and the Reference Diet

**Figure 3:**
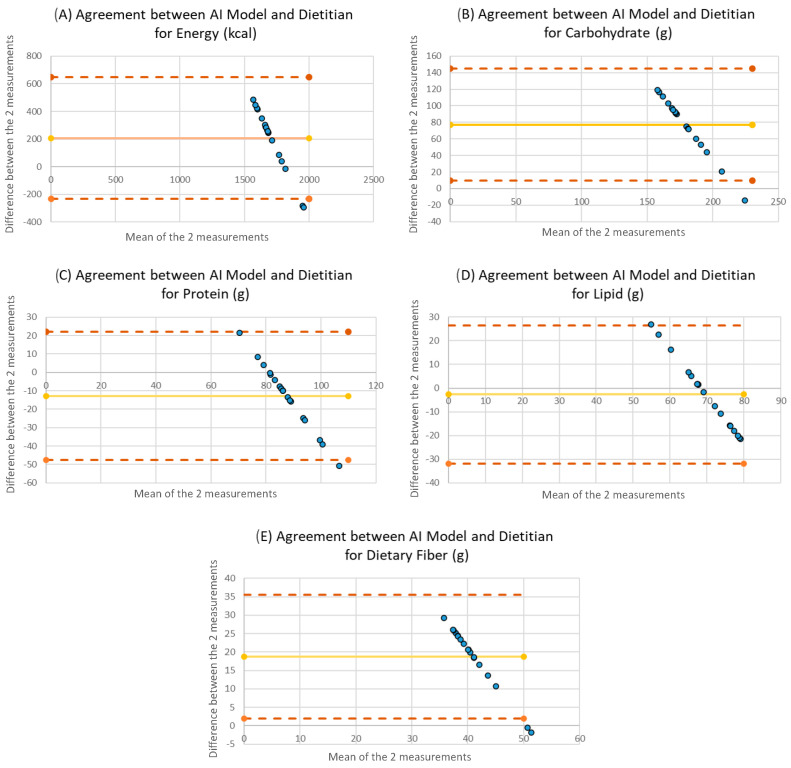
Bland–Altman analysis of fits and differences between diets generated by language models and reference diets. Panels show agreement for (**A**) energy, (**B**) carbohydrate, (**C**) protein, (**D**) lipid, and (**E**) dietary fiber. The solid line represents the mean difference (bias), and the dashed lines indicate the 95% limits of agreement.

Agreement between the three-day dietary plans generated by the LLMs and the reference diet was assessed using Bland–Altman analysis, with the corresponding plots presented in Figure 3. For energy intake, the mean difference relative to the reference diet was 207.7 kcal, with 95% LOA ranging from −233.6 to 649.0 kcal, indicating a systematic tendency of AI-generated diets to provide lower total energy compared with the reference diet. Considering a clinically relevant threshold of ±10% deviation from the target energy intake (180 kcal for an 1800 kcal diet), several AI-generated plans exceeded this range, indicating potential clinical implications for glycemic management and dietary planning. Across energy and macronutrient components, the proportion of observations falling outside the 95% limits of agreement ranged from 5.6% to 11.1%. The proportions of observations outside the 95% LOA for energy and macronutrients are presented in Supplementary Table S2.

The mean differences and limits of agreement for individual macronutrients were as follows:

Carbohydrate, +77.1 g (95% LOA: 9.6 to 144.6 g); protein, −12.7 g (95% LOA: −47.6 to 22.0 g); fat, −2.7 g (95% LOA: −31.9 to 26.5 g); and dietary fiber, +9.6 g (95% LOA: −7.2 to 26.4 g).

Overall, Bland–Altman plots demonstrated the presence of observations outside the limits of agreement for all evaluated nutrients, reflecting systematic discrepancies between AI-generated dietary plans and the dietitian-designed reference diet, particularly for energy and carbohydrate content.

### 3.4. Dietitian-Based Evaluation of Guideline Adherence

Dietitian-based evaluations of the three-day dietary plans generated by the LLMs are presented in Table 3. Inter-rater reliability between the two dietitians was assessed using the ICC, which indicated a high level of agreement (ICC = 0.806; 95% confidence interval: 0.654–0.896; *p* < 0.001).

Across evaluation domains, diets generated by Claude Sonnet 4.5 consistently received higher scores for guideline adherence, appropriateness of meal distribution, and accuracy of reported energy and macronutrient values. In contrast, Gemini 2.5 Flash and Gemini 2.5 Pro received lower scores across most evaluation criteria, particularly in guideline adherence, meal distribution, and consistency between stated and calculated nutrient values. ChatGPT-5 variants demonstrated intermediate performance, with relatively higher scores for clarity of dietary recommendations but greater variability across evaluative domains.

Sensitivity analyses using linear mixed-effects models showed negligible random intercept variance for most nutrients, indicating minimal clustering of daily outputs within individual models. Intraclass correlation coefficients ranged from −0.073 to 0.642, suggesting low to moderate similarity across daily diets generated by the same model (Supplementary Tables S4 and S5).

### 3.5. Micronutrient Reporting

Although the standardized prompt explicitly requested micronutrient values, a systematic review of model outputs revealed that most LLMs reported only target or average values for selected micronutrients (most commonly sodium and calcium) rather than day-specific calculated amounts. Day-level micronutrient data, which would be required for a valid quantitative comparison analogous to the macronutrient analyses, were not provided by any model. Consequently, a systematic micronutrient adequacy analysis was not feasible, and this finding is itself reported as a qualitative limitation of current LLM outputs in clinical nutrition practice.

## 4. Discussion

Advances in artificial intelligence technologies have led to a rapid expansion of AI applications across various fields, including nutrition and dietetics. AI-based tools for providing nutrition advice and generating dietary plans have become an increasingly discussed topic in both academic and clinical contexts with the widespread adoption of LLMs [20]. Diabetes is one of the most frequently discussed disease conditions in interactions with LLMs. LLMs are used in diabetes management for purposes such as meal planning, providing personalized recipe recommendations, and estimating insulin doses [21]. Nevertheless, individualized MNT, planned and monitored by a RD, remains an integral component of glycemic control and the management of T2DM [22]. The increasing use of LLMs in diabetes management highlights the need to validate the reliability and clinical appropriateness of the recommendations provided by these models.

In national and international guidelines, the primary emphasis in the medical nutrition therapy of T2DM is placed not on macronutrient distribution percentages, but rather on food quality, degree of processing, dietary diversity, and sustainability [3]. Moreover, systematic reviews and meta-analyses published in recent years indicate that high adherence to healthy dietary patterns is associated with significant improvements in HbA_1c_, fasting plasma glucose, and cardiovascular risk factors [23,24]. In this study, diets for T2DM generated by commonly used LLMs were compared with a reference diet prepared by a dietitian working in a diabetes outpatient clinic in accordance with current diabetes guidelines, in terms of energy and macronutrient content, and the adherence of AI-generated diets to these guidelines was evaluated.

The results suggest that LLMs can generate structured dietary plans for diabetes when provided with a standardized prompt; however, notable limitations remain regarding quantitative accuracy and clinical relevance. In this study, the diets produced by LLMs did not meet the specified energy targets and generally provided lower energy compared with the reference diet prepared by a dietitian. Diets generated by ChatGPT-5 Auto, Gemini 2.5 Flash, and Gemini 2.5 Pro contained less energy than requested and showed poor alignment with the reference diet. By contrast, the diet generated by the Claude Sonnet 4.5 model was closer to both the prescribed energy level and the reference diet. Consistent with these findings, previous research evaluating AI-generated diets designed for weight management across different sexes and energy requirements reported that, while overall diet quality was acceptable, macronutrient distribution was often inappropriate. In that study, consistent with the findings of this study, AI-generated diets, including those produced by Gemini 2.5. and ChatGPT-4, exhibited deviations in energy content ranging from 5% to 20% [25]. In this study, differences in macronutrient distribution were also observed among the models. The diet generated by Claude Sonnet 4.5 showed the closest alignment with the reference diet in terms of both energy and dietary fiber content. These findings suggest that factors such as LLM architecture, the scope of training data, and the way instructions are interpreted may influence diet quality, leading to clinically meaningful performance differences among language models in the context of clinical nutrition.

In the diets generated by LLMs, the percentage of energy derived from carbohydrates does not align with the range of 45–60% recommended by diabetes nutrition guidelines [1,3,17,24] (Supplementary Table S1). Deviations in carbohydrate content showed a systematic reduction in the Gemini 2.5 Flash and Gemini 2.5 Pro models. Consistent with our findings, the study conducted by Bayram et al. reported that AI-generated diets frequently exhibit carbohydrate contents that are not aligned with current dietary guidelines [7]. This tendency toward lower carbohydrate content in AI-generated diets may be related to the increasing popularity of low-carbohydrate dietary approaches in recent years.

Current guidelines recommend increasing dietary fiber intake primarily through natural sources such as whole grains, legumes, vegetables, fruits, and nuts, while limiting dietary patterns rich in refined carbohydrates and low in fiber [3,17,24]. In the ADA Standards of Care in Diabetes 2026, the importance of high-quality, minimally processed, and fiber-rich carbohydrate sources is further emphasized, regardless of the absolute amount of carbohydrate included in the meal plan [3]. In the present study, all AI models except Claude Sonnet 4.5 generated diets containing lower amounts of dietary fiber compared with the reference diet. Similarly, a study comparing AI-generated healthy diets with dietitian-designed diets reported that the reference diet had a higher carbohydrate content, was more compliant with the prompt, and was richer in dietary fiber, consistent with our findings [26]. Overall, the low dietary fiber content of AI-generated diets may be considered a major limitation to the ability of LLMs to produce reliable and guideline-adherent nutrition plans for individuals with T2DM. From a clinical perspective, interpretation of agreement between AI-generated diets and the reference diet should also consider meaningful nutritional thresholds. For example, deviations of approximately ≥ 10% in daily energy intake may influence glycemic control and weight management in individuals with type 2 diabetes. Similarly, dietary fiber intake recommendations suggest approximately 14 g per 1000 kcal, which corresponds to roughly 25–30 g/day for an 1800 kcal diet. Some AI-generated diet plans showed deviations from these targets, which may have potential implications for long-term metabolic outcomes.

In our study, a more heterogeneous distribution in protein content was observed across models, with several models producing diets higher in protein than the reference diet. Similarly, a study investigating the reliability and accuracy of diets generated by LLMs reported deviations of up to ±65 g in protein content between different models and found that LLMs were unable to consistently achieve appropriate macronutrient distributions [27]. This finding suggests that the increasing use of LLMs may lead to recommendations that are incompatible with diabetes-related complications such as nephropathy and underscores the need for individualized clinical evaluation in the presence of comorbid conditions accompanying diabetes.

In this study, the Claude Sonnet 4.5 model systematically generated diets with higher fat content, whereas the Gemini 2.5 Flash model produced diets with lower fat content. In a study by Bayram et al., which evaluated energy calculations, nutrient distribution, and adherence to nutrition care process standards using 24 different virtual patient profiles, ChatGPT-4 generated diets with energy contents closest to the target; however, all included AI models were found to produce diets with excessively high fat content [7]. In a study evaluating diets generated by ChatGPT-4 for dyslipidemia and hypertension, the model was reported to perform well in providing general health recommendations; however, it produced diets characterized by low carbohydrate (24%) and high fat (54%) content. Additionally, despite successful recommendations, the diets were found to be high in sodium, calcium, and cholesterol. Consistent with our findings, these results confirm that while LLMs may provide disease-appropriate recommendations, they can generate incongruent outputs in actual diet planning. The same study also reported that when a fixed energy target was provided to the language model, it produced diets more closely aligned with the specified energy level compared with prompts without an explicit energy target [28]. However, in our study, LLMs were unable to generate diets consistent with the specified energy target despite the provision of an explicit energy value. Systematic discrepancies were observed between LLM-generated diets and the reference diet for both energy and macronutrients. In particular, the wide limits of agreement observed for energy and carbohydrate intake indicate that AI-generated outputs should be interpreted with caution in individual clinical practice. Although some LLMs were able to produce diets with macronutrient distributions close to the reference diet, inconsistencies in energy and nutrient content, together with the lack of consideration of individualized requirements and comorbidities, demonstrate that AI-based dietary plans do not yet provide a level of accuracy and appropriateness sufficient to replace dietitian-guided nutrition therapy.

In a study comparing patient education materials generated by expert physicians and LLMs for diabetic kidney disease, some LLMs were found to produce materials of similar quality to those prepared by physicians; however, models such as ChatGPT-4 were reported to be insufficient in terms of understandability and accuracy [29]. Similarly, in the study conducted by Bayram et al. using T2DM patient profiles, recommendations related to complications such as hypoglycemia were found to be insufficient [7]. Therefore, consistent with the findings of our study, it is emphasized that health-related recommendations generated by LLMs should always be reviewed and validated by qualified healthcare professionals [7,29]. In a study evaluating the adherence of dietary recommendations for heart health generated by different LLMs to scientific guidelines, the models were found to provide generally appropriate and reliable advice; however, their recommendations regarding quantitative values, such as carbohydrate and sugar intake, were reported to be weak [30]. In our study, although the importance of dietary fiber intake was frequently mentioned textually in the nutrition recommendations generated by LLMs, the majority of AI-generated diets did not adequately and consistently include fiber-rich food groups. This finding indicates that while LLMs are strong in generating explanatory recommendations, they remain limited in translating these suggestions into quantitative and food-based dietary planning. A further notable finding was the failure of most LLMs to provide complete micronutrient data despite explicit prompting. This is clinically relevant in T2DM, as deficiencies in key micronutrients such as magnesium, vitamin D, and zinc have been associated with impaired glycemic control, representing an additional gap in the clinical utility of current LLM outputs [31,32].

Although meal-level macronutrient distributions were not analyzed in detail in this study, the low dietitian evaluation scores observed for some models suggest that AI-generated diets may have limitations not only in terms of total daily macronutrient targets but also regarding meal structure and overall daily planning consistency. This finding indicates that while LLMs may demonstrate a certain level of adequacy in calculating total nutrient values, they remain insufficient in reflecting meal planning, which is a key component of MNT. Current diabetes guidelines emphasize that one of the most important components of MNT is individualization. The individualized approach prioritizes nutrition therapy that takes into account personal characteristics, clinical findings, and lifestyle factors rather than applying uniform dietary recommendations [3,17,24]. From a clinical perspective, these findings suggest that large language models should not currently be used as standalone tools for medical nutrition therapy in T2DM. Instead, they may serve as supportive tools within supervised clinical workflows, where dietitians critically evaluate and adapt AI-generated recommendations. The systematic deviations observed in energy and macronutrient composition highlight the need for guideline-informed model development, domain-specific training datasets, and rigorous clinical validation before such tools can be safely integrated into routine nutrition practice.

## 5. Strengths and Limitations

This study has several strengths. All LLMs were tested using the same standardized prompt in independent sessions, allowing a controlled comparison across models. In addition, AI-generated meal plans were compared with a reference diet developed by an expert dietitian on the basis of current evidence-based recommendations for T2DM, which provided a clinically meaningful benchmark. Another strength is that the study combined quantitative nutrient analysis with independent expert scoring of guideline adherence, enabling both analytical and clinical interpretation of LLM-generated outputs.

Nevertheless, some limitations should be acknowledged. The analysis was limited to a single standardized 3-day, 1800 kcal T2DM scenario, and therefore the findings may not be directly generalized to other dietary prescriptions or more complex clinical contexts. In weight-loss scenarios with lower energy targets, macronutrient distribution patterns may differ substantially from those observed in this study. More critically, in renal-modified diets required for patients with diabetic nephropathy, protein restriction and electrolyte management represent essential safety parameters; given the inter-model variability in protein content identified in the present study, the clinical risk associated with LLM-generated dietary plans may be considerably greater in such contexts. The number of evaluated LLMs was also limited to those accessible during the study period, and because the performance of such systems changes rapidly over time, the results should be interpreted as a time-specific assessment. Despite explicit instructions to include micronutrient data, most LLMs failed to provide day-specific calculated values, reporting only general targets or averages for selected nutrients, which precluded a systematic quantitative micronutrient analysis. The study therefore primarily focused on macronutrients and dietary fiber as core components of T2DM meal planning. Future research should examine a broader range of nutrients and clinical scenarios, including weight-loss and renal-modified dietary contexts, more diverse patient profiles, and newly released models.

## 6. Conclusions

This study demonstrates that current large language models generate dietary plans for T2DM that show systematic deviations from guideline-based reference diets. In our study, AI-generated diets largely exhibited similar energy and macronutrient profiles and did not incorporate adaptations based on individual characteristics. LLMs appeared to treat individuals with T2DM largely as a homogeneous group. In contrast, clinical factors such as sex, age, body weight, and comorbid conditions can substantially influence individual energy and protein requirements. Despite this, when provided with a standardized prompt, LLMs did not request additional information to support individualization and failed to deliver personalized dietary recommendations. This contradicts the principle of individualized nutrition therapy and underscores that dietitian guidance remains essential in the management of T2DM. Nevertheless, in an evolving digital landscape, the presence of LLMs is undeniable, and they may be utilized as supportive tools by dietitians within MNT.

## Figures and Tables

**Table 1:** Guidance data on lifestyle management in T2DM.

Foundation	Recommendations
IDF [1]	Individualized energy intake based on body weight and metabolic status; emphasis on diet quality; limitation of refined carbohydrates, sugar-sweetened beverages, and ultra-processed foods; preference for healthy fat sources; promotion of fiber-rich foods.
SEMT [17]	Individualized energy prescription; macronutrient distribution within recommended ranges (carbohydrate 45–60%, protein 10–20%, fat 20–35%); minimum carbohydrate intake of 130 g/day; saturated fat < 7–8%, trans fat < 1%; dietary fiber ≥14 g/1000 kcal; promotion of regular physical activity, recommending at least 150 min per week of moderate-intensity aerobic exercise, along with minimizing sedentary behavior.
ADA [3]	Individualized energy intake with no fixed caloric target, based on age, sex, body weight, physical activity level, and metabolic status. Carbohydrate intake may account for approximately 45–60% of total energy and should be adjusted according to individual glycemic response and tolerance. Protein intake is recommended at 15–20% of total energy. No specific total fat range is defined; however, saturated fat intake should be minimized, trans fat intake should be avoided, and mono- and polyunsaturated fatty acids should be preferred. Dietary fiber intake should be at least 14 g per 1000 kcal, with emphasis on whole grains, legumes, vegetables, and whole fruits. Regular physical activity is recommended, including at least 150 min per week of moderate-intensity aerobic activity, and avoidance of prolonged sedentary behavior (≥30 min of uninterrupted sitting).
ADA-EASD Consensus Report [2]	No fixed macronutrient distribution; individualized and sustainable dietary patterns; emphasis on high dietary fiber intake and overall diet quality; increased intake of whole grains, legumes, fruits, and vegetables; encouragement of regular physical activity and reduced sedentary time.

IDF: International Diabetes Federation; SEMT: Society of Endocrinology and Metabolism of Turkey; ADA: American Diabetes Association; ADA-EASD: American Diabetes Association–European Association for the Study of Diabetes.

**Table 2:** Comparison of energy and nutrient content of large language models and reference diet.

	ChatGPT-5 Instant	ChatGPT-5 Thinking	ChatGPT-5 Auto	Claude Sonnet 4.5	Gemini 2.5 Flash	Gemini 2.5 Pro	Reference Diet
Energy (kcal)	1617.1 ± 179.8	1572.0 ± 192.8	**1567.3 ± 26.8**	1973.0 ± 216.8	**1412.8 ± 123**	**1466.2 ± 76.4**	1809.1
CHO (g)	**142.5 ± 5.0**	**131.2 ± 21.7**	**137.3 ± 23.7**	195.1 ± 37.1	**119.4 ± 24**	**115.8 ± 13.2**	217.4
Protein (g)	**103.4 ± 5.6**	**91.9 ± 2.4**	80.4 ± 2.7	111.5 ± 13.2	98.6 ± 30.2	77.9 ± 16	81.2
Fat (g)	**68.6 ± 6.6**	73.1 ± 24	73.1 ± 24	79.7 ± 11.6	57.5 ± 19.4	74.3 ± 10.6	68.3
Fiber (g)	**29.0 ± 3.3**	**27.5 ± 2.5**	**30.1 ± 3.4**	46.6 ± 8.6	31.9 ± 7.6	**24.6 ± 3.5**	41.3

Bold values indicate statistically significant differences compared with the reference diet (paired-sample *t*-test, *p* < 0.05).

**Table 3:** Dietitian-based evaluation scores of AI-generated dietary plans according to guideline adherence criteria.

	ChatGPT-5 Auto		ChatGPT-5 Instant		ChatGPT-5 Thinking		Claude Sonnet 4.5		Gemini Flash 2.5		Gemini Pro 2.5	
	RD 1	RD 2	RD 1	RD 2	RD 1	RD 2	RD 1	RD 2	RD 1	RD 2	RD 1	RD 2
Did the AI model give clear and correct dietary recommendations next to the diet?	6	7	10	9	6	7	10	10	6	4	7	7
Did the AI model prepare a diet that complied with national and international guidelines?	5	4	8	7	5	5	9	8	3	3	4	4
Was the AI model able to distribute meals in accordance with diabetes?	6	5	5	6	2	4	9	8	2	4	3	4
Was the amount of energy suggested by the AI model in line with what was desired?	6	3	5	5	6	4	9	10	3	3	4	3
Did the diet suggested by the AI model provide enough protein?	7	4	4	5	7	5	6	9	7	4	5	4
Were the amounts of energy and macronutrients indicated by the AI model for the diet it prepared compatible with the actual evaluation?	4	3	5	4	4	4	9	10	2	2	2	3

Scores range from 1 to 10, with higher scores indicating greater adherence to diabetes nutrition guidelines.

## Data Availability

The data presented in this study are available from the corresponding author upon reasonable request.

## Supplementary Materials

The following supporting information can be downloaded at: https://www.mdpi.com/article/10.3390/healthcare14060739/s1, Table S1. Percentage energy contribution from protein, fat, and carbohydrate in AI-generated diet plans. Table S2. Bland–Altman Numerical Outputs for Energy and Macronutrients. Table S3. Proportion of observations falling outside the 95% limits of agreement (LOA) for energy and macronutrients. Table S4. Linear mixed-effects model sensitivity analysis accounting for clustering of daily observations within each large language model. Table S5. Intraclass correlation coefficients (ICCs) assessing within-model similarity across daily diet plans generated by each large language model. Figure S1. High-Resolution Bland–Altman Plots for All Macronutrients. Figure S2. High-Resolution Box Plot for All Macronutrient and Dietary Fiber. Text S1. Full Prompt Script Used For AI Diet Generation. Text S2. Full Version of Reference Diet and AI-Generated Diets.

## Abbreviations

The following abbreviations are used in this manuscript:

T2DM	Type 2 Diabetes Mellitus
MNT	Medical Nutrition Therapy
AI	Artificial Intelligence
LLMs	Large Languages Models
IDF	International Diabetes Federation
SEMT	Society of Endocrinology and Metabolism of Turkey
ADA	American Diabetes Association
EASD	European Association for the Study of Diabetes
LOA	Limits of Agreement
ICC	Intraclass Correlation Coefficient
RD	Registered Dietitian
