# Supplementary material for: Large Language Models as Clinical Nutrition Decision Tools: Quantitative Bias and Guideline Deviation in Type 2 Diabetes Meal Planning
Source: Healthcare (Basel). 2026 Mar 13;14(6):739. doi: 10.3390/healthcare14060739 (PMC13026456; doi:10.3390/healthcare14060739)
Supplement: Supplementary file 1 [file healthcare-14-00739-s001.zip › healthcare-4182999-supplementary.pdf]

Supplementary Materials

1. Supplementary Figures, Tables and Texts

1.1. Supplementary Tables

This table presents the distribution of daily energy derived from macronutrients in three-day dietary plans generated by each artificial intelligence model for individuals with type 2 diabetes. While the main result tables report the three-day mean values of total energy, carbohydrate, protein, fat, and dietary fiber of the prepared diets, this supplementary table aims to illustrate differences in macronutrient energy distribution across the dietary plans. The diet designed by the registered dietitian reflects macronutrient ranges consistent with current dietary guidelines. In the dietitian-designed reference diet, the distribution of total energy from macronutrients was 48.0% from carbohydrates, 17.9% from protein, and 33.9% from fat.

Table S1. Percentage energy contribution from protein, fat, and carbohydrate in AI-generated diet plans.

		ChatGPT-5 Auto	ChatGPT-5 Instant	ChatGPT-5 Thinking	Claude Sonnet 4.5	Gemini Flash 2.5	Gemini Pro 2.5
**Carbohydrate (%)**	**Day 1**	32.8	33.6	27.2	36.5	29.5	33.1
	**Day 2**	30.3	33.5	32.3	37.6	42.8	26.1
	**Day 3**	42.2	38	41.8	44	29.7	36.2
**Protein (%)**	**Day 1**	21.6	27.9	23	27.4	23.5	24.4
	**Day 2**	19.1	25.7	21.4	23	21.4	22.1
	**Day 3**	20.9	23.5	26.3	18.3	39.9	17.1
**Fat (%)**	**Day 1**	39.5	37.3	48.4	34.7	45.8	41.1
	**Day 2**	49.8	40	45.7	38.1	34.4	50.3
	**Day 3**	35.5	37.5	29.6	36.1	28.2	45.2

This table contains the entire numerical output of the Bland–Altman analysis that evaluates the agreement between the methods (bias, standard deviation, lower and upper LOA limits, point ratio outside the LOA).

Table S2. Bland–Altman Numerical Outputs for Energy and Macronutrients.

	Bias	SD	Lower LOA	Upper LOA	Observations outside the LOA
**Energy (kcal)**	207.68	225.18	-233.66	649.04	2/18 (11.1%)
**Carbohydrate (g)**	77.15	34.45	9.6	144.69	1/18 (5.6%)
**Protein (g)**	-12.79	17.7	-47.65	22.06	1/18 (5.6%)
**Fat (g)**	-2.7	14.9	-31.91	26.5	1/18 (5.6%)
**Dietary fiber (g)**	18.74	8.59	1.89	35.59	2/18 (11.1%)

LOA= Limit of Agreement. Bias = mean difference between the dietitian-designed reference diet and AI-generated diets.

Since the three-day dietary plans generated by a total of six different versions of three large language models were evaluated in the study, 18 distinct observations were obtained. Under these conditions, it was determined that 11.1% of the observed values for energy, 5.6% for carbohydrate, 5.6% for protein, and 11.1% for dietary fiber fell below the lower limit of agreement. In contrast, for dietary fat intake, 5.6% of the observations exceeded the upper limit of agreement.

Table S3. Proportion of observations falling outside the 95% limits of agreement (LOAs) for energy and macronutrients.

Nutrient	Total Comparisons (n)	Outside LOAs (n)	Outside LOAs (%)
**Energy (kcal)**	18	2	11.1
**Carbohydrate (g)**	18	1	5.6
**Protein (g)**	18	1	5.6
**Fat (g)**	18	1	5.6
**Dietary Fiber (g)**	18	2	11.1

LOA: Limit of agreement.

To evaluate whether daily dietary outputs generated by the same language model could introduce clustering effects, linear mixed-effects models were fitted with model identity included as a random intercept. The results demonstrate negligible random-effect variance for most nutrients, suggesting minimal within-model clustering. These findings support the analytical assumption used in the primary analyses that daily diets generated by the same language model can reasonably be treated as independent observations.

Table S4. Linear mixed-effects model sensitivity analysis accounting for clustering of daily observations within each large language model.

Outcome	Fixed effect (intercept) F-test	p	Random effect variance
**Energy deviation**	15.312	0.001	0.000
**Carbohydrate deviation**	90.226	<0.001	0.000
**Protein deviation**	9.317	0.007	0.000
**Fat deviation**	0.059	1.000	111.049
**Dietary fiber deviation**	22.662	<0.001	0.000

To further assess whether daily dietary plans generated by the same language model could be considered independent observations, intraclass correlation coefficients (ICCs) were calculated using a two-way random-effects model with absolute agreement. Single-measure ICC values were reported because each daily diet plan was treated as an individual observation in the primary analyses.

Table S5. Intraclass correlation coefficients (ICCs) assessing within-model similarity across daily diet plans generated by each large language model.

Nutrient	ICC (Single measure)	95% CI	p
**Energy (kcal)**	0.566	0.050–0.914	0.02
**Carbohydrate (g)**	0.507	0.057–0.891	0.014
**Protein (g)**	0.261	−0.268–0.819	0.180
**Fat (g)**	−0.073	−0.332–0.556	0.591
**Dietary fiber (g)**	0.642	0.193–0.930	0.003

1.2. Supplementary Figures

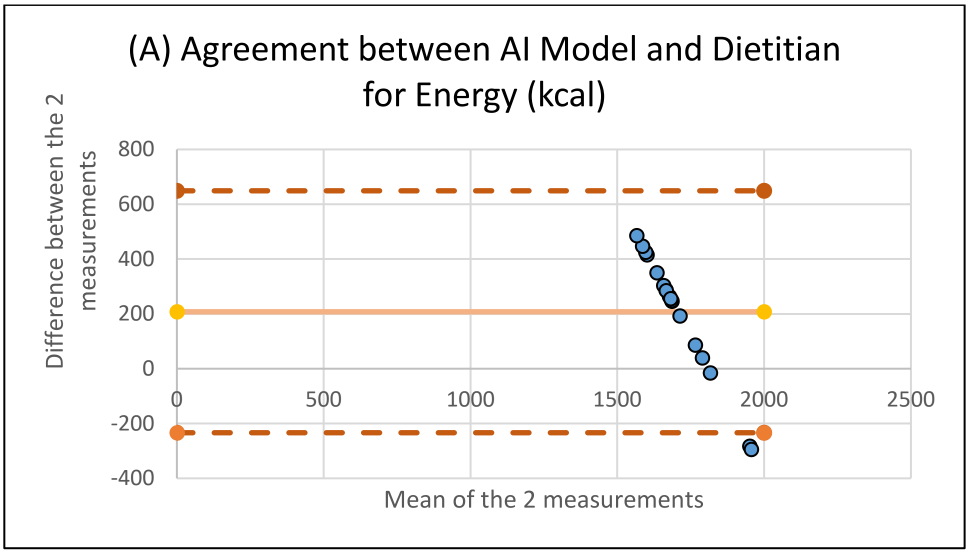

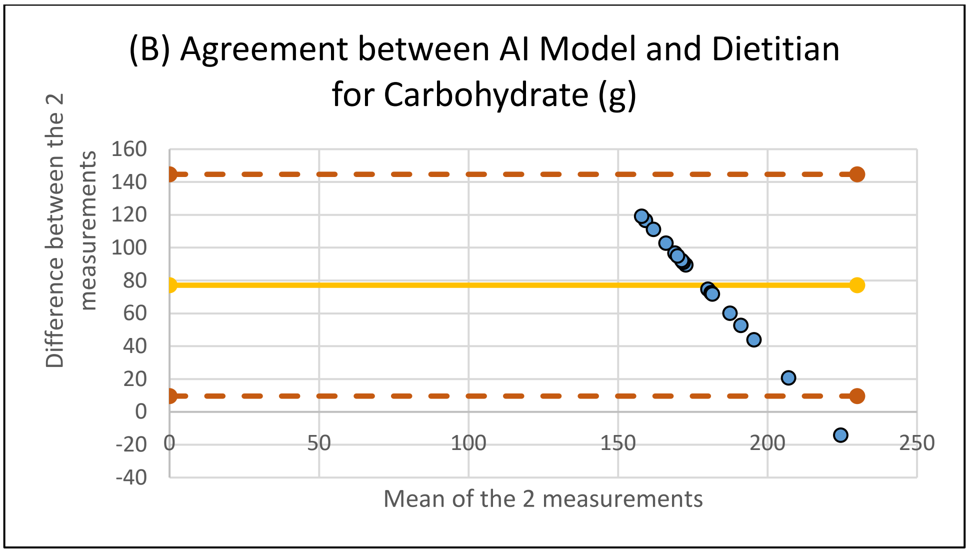

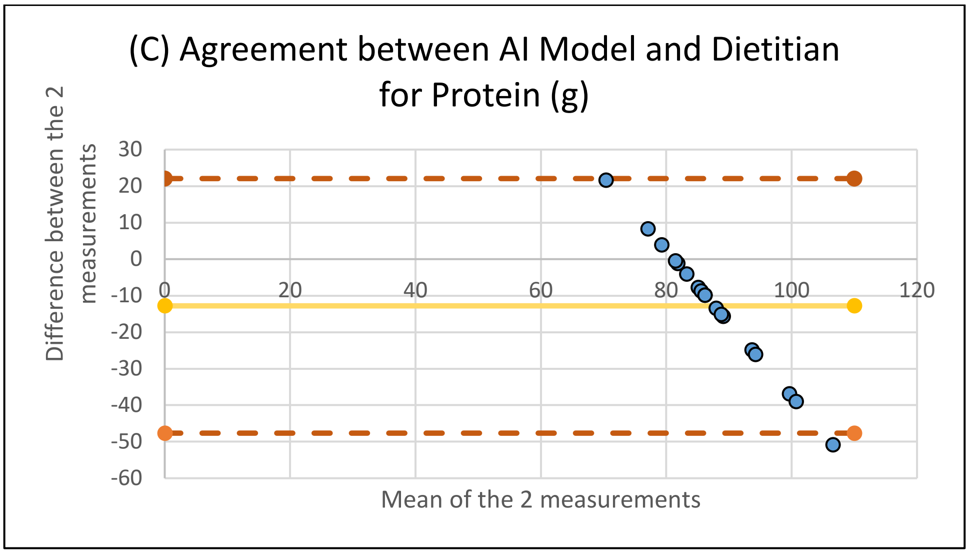

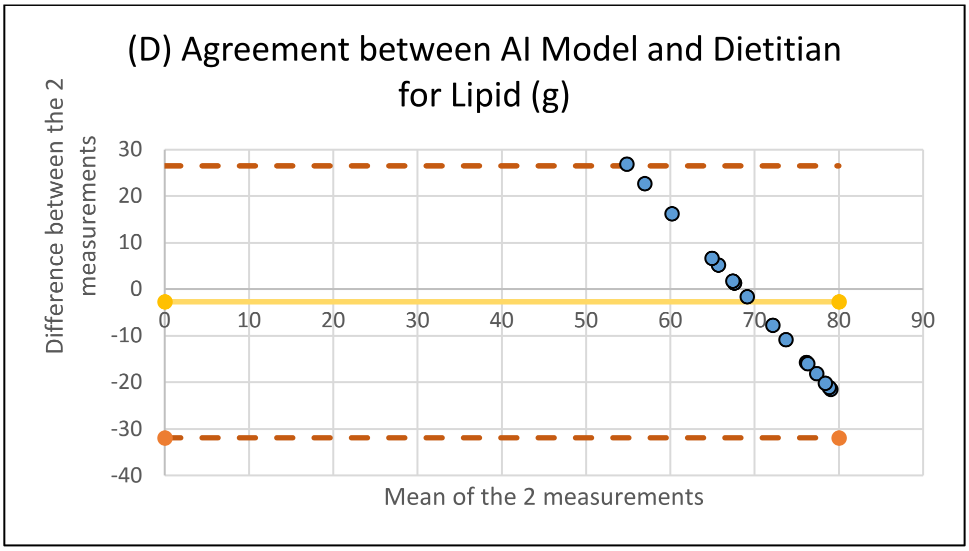

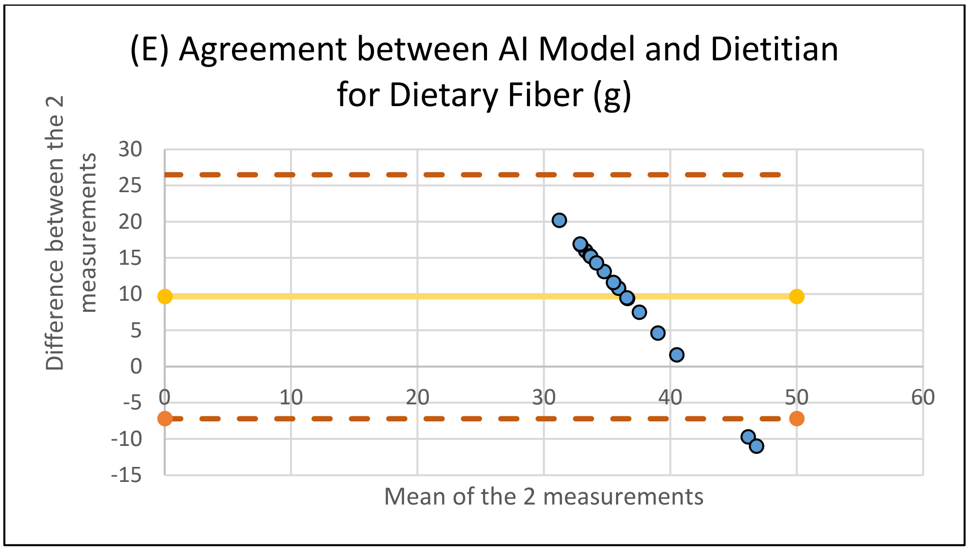

Supplementary Figure S1. High-Resolution Bland–Altman Plots for All Macronutrients. High-resolution single-panel versions of the multi-panel Bland–Altman graph included in the main text are presented separately for energy, protein, fat, and carbohydrate.

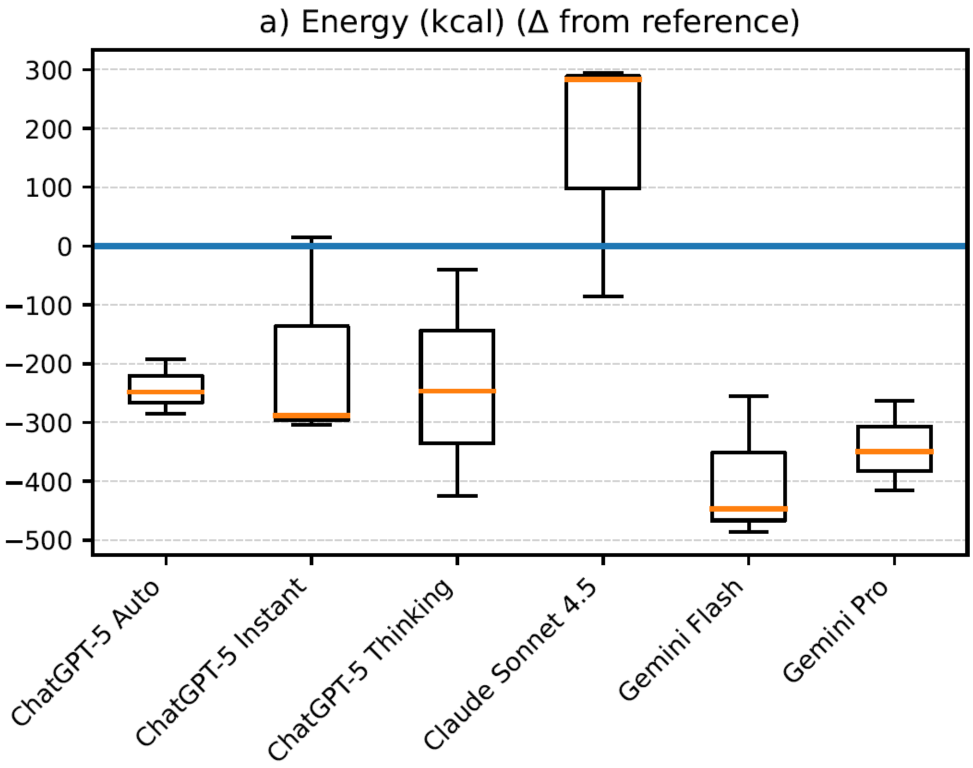
a) Energy (kcal) (Δ from reference)
300
200
100
0
−100
−200
−300
−400
−500
ChatGPT-5 Auto
ChatGPT-5 Instant
ChatGPT-5 Thinking
Claude Sonnet 4.5
Gemini Flash
Gemini Pro

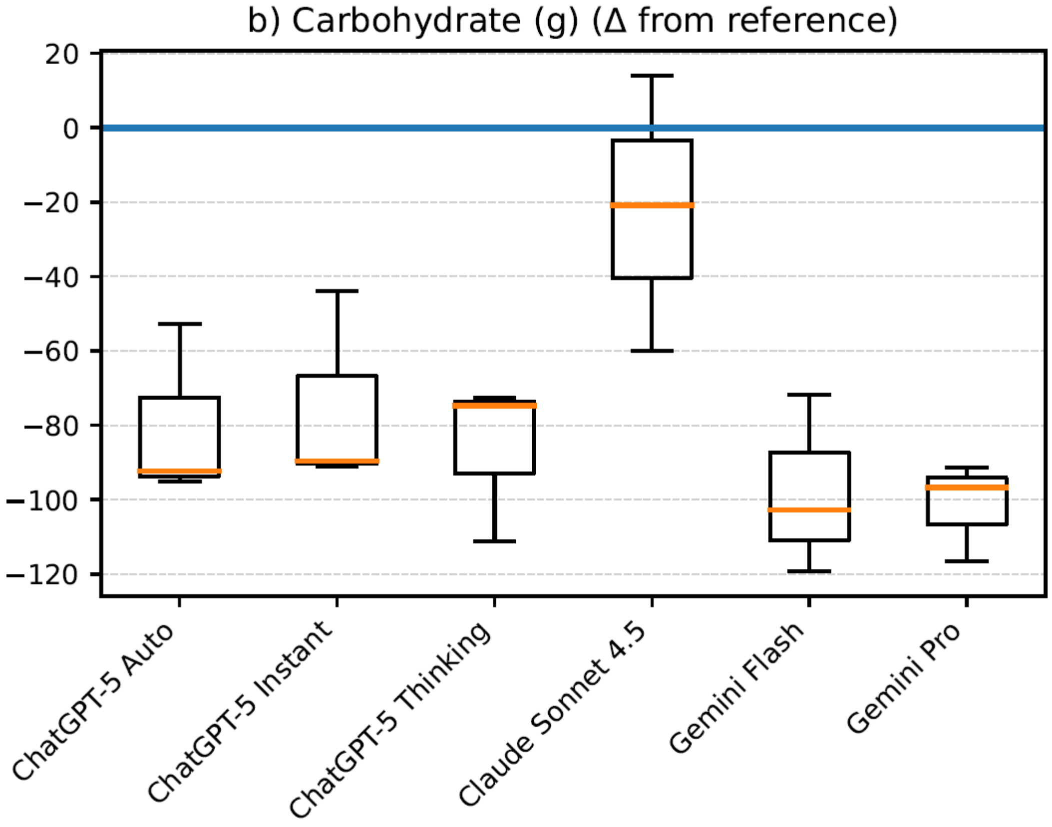

b) Carbohydrate (g) (Δ from reference)
20
0
−20
−40
−60
−80
−100
−120
ChatGPT-5 Auto
ChatGPT-5 Instant
ChatGPT-5 Thinking
Claude Sonnet 4.5
Gemini Flash
Gemini Pro

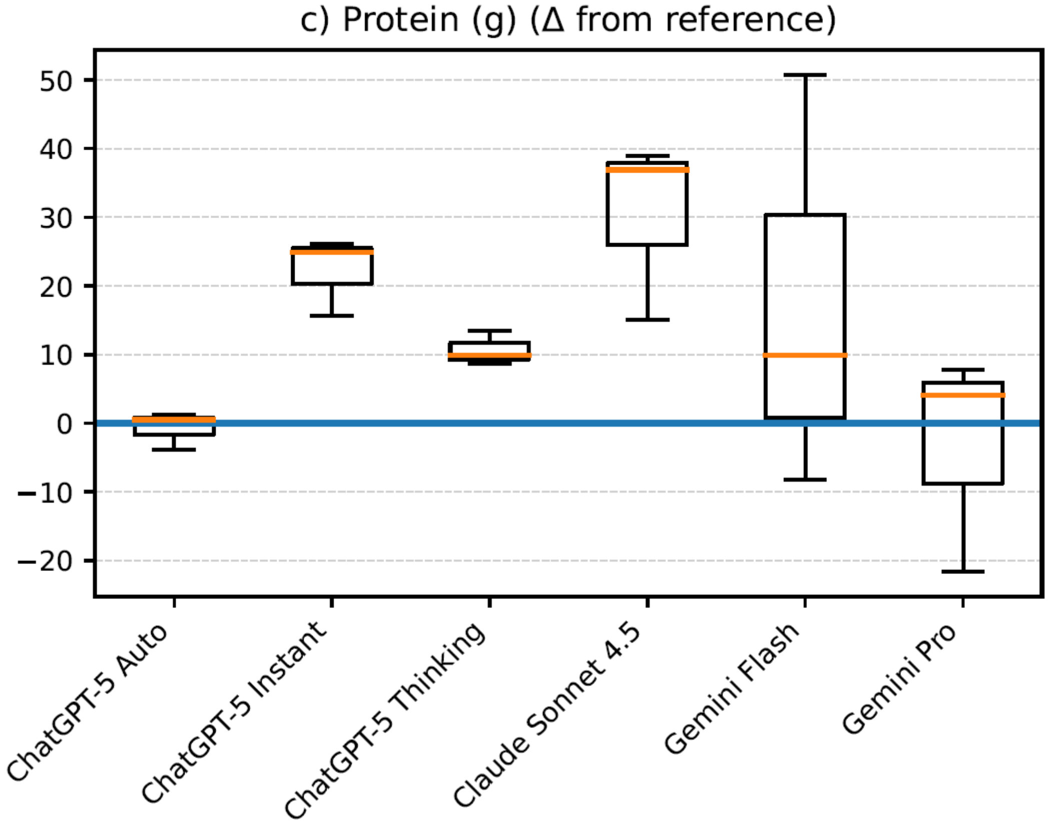

c) Protein (g) (Δ from reference)
50
40
30
20
10
0
−10
−20
ChatGPT-5 Auto
ChatGPT-5 Instant
ChatGPT-5 Thinking
Claude Sonnet 4.5
Gemini Flash
Gemini Pro

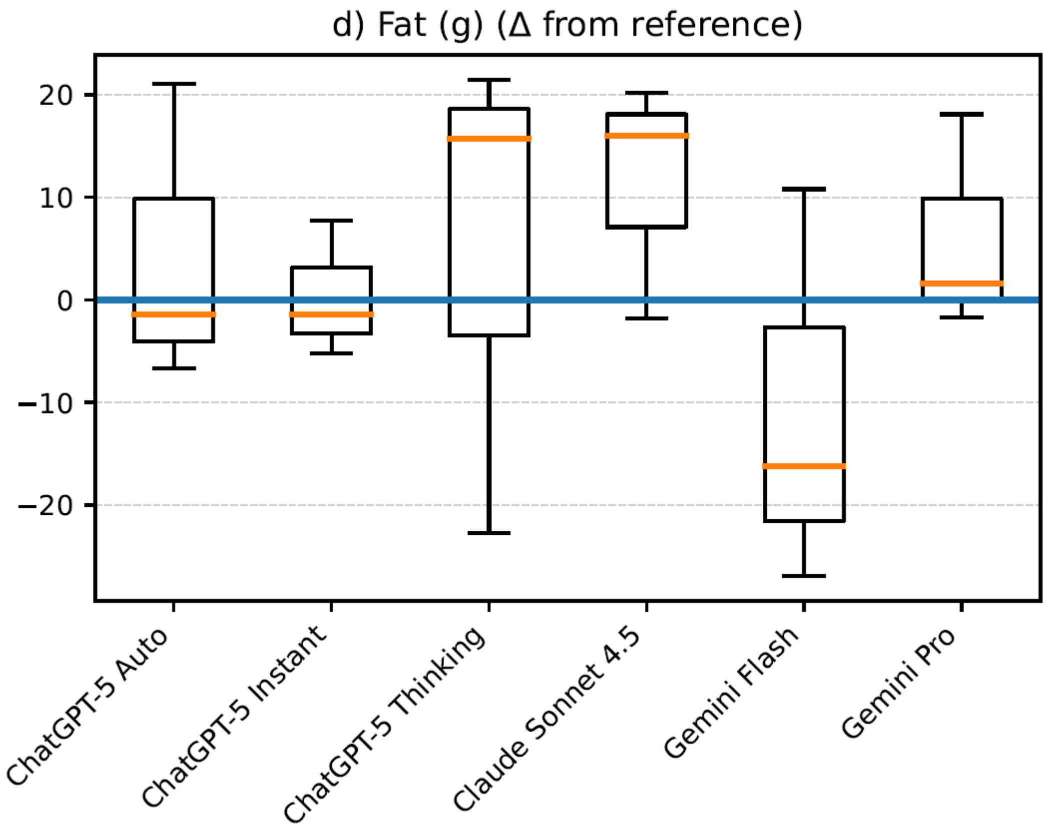

d) Fat (g) (Δ from reference)
20
10
0
−10
−20
ChatGPT-5 Auto
ChatGPT-5 Instant
ChatGPT-5 Thinking
Claude Sonnet 4.5
Gemini Flash
Gemini Pro

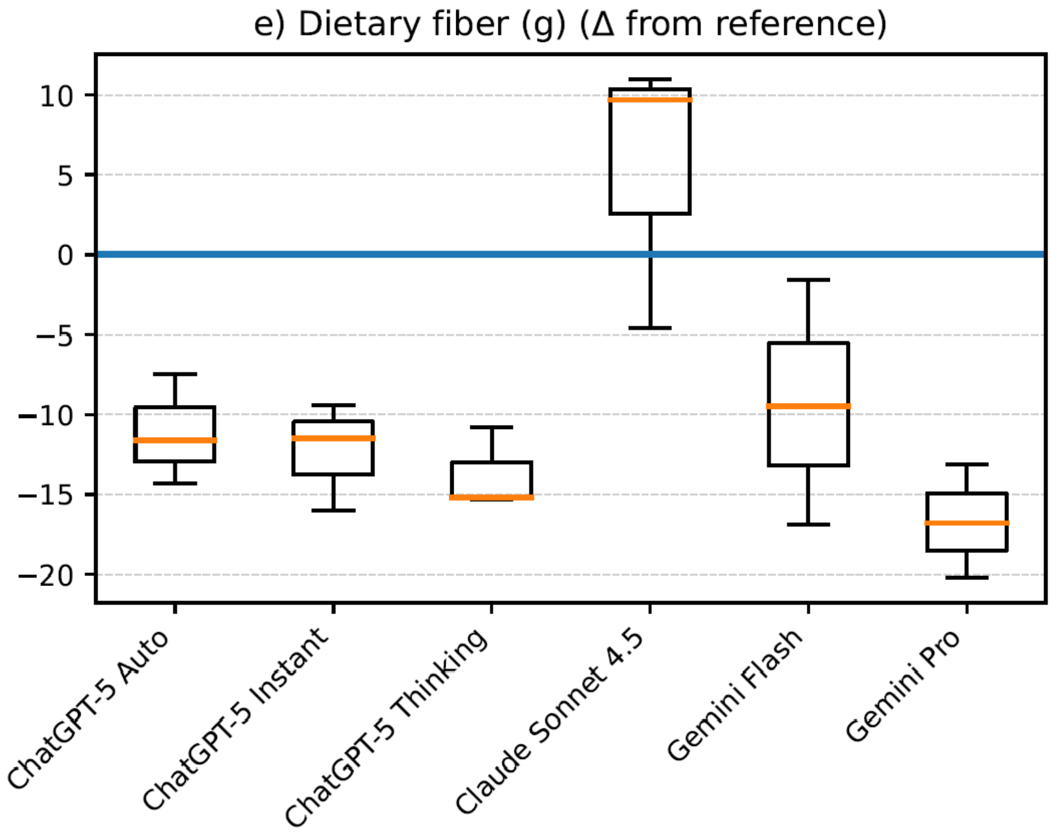

e) Dietary fiber (g) (Δ from reference)
10
5
0
−5
−10
−15
−20
ChatGPT-5 Auto
ChatGPT-5 Instant
ChatGPT-5 Thinking
Claude Sonnet 4.5
Gemini Flash
Gemini Pro

Supplementary Figure S2. High-Resolution Box Plot for All Macronutrient and Dietary Fiber. High-resolution single-panel versions of the multi-panel Box Plot graph included in the main text are presented separately for energy, carbohydrate, protein, fat and dietary fiber.

1.3. Supplementary Texts

Supplementary Text S1. Full Prompt Script Used For AI Diet Generation

In this section, the full text of the standard prompt used to guide the large language models is provided.

- *Prepare a 3-day 1800 kcal diet plan for individuals with type 2 diabetes based on national and international nutrition guidelines. Also include the macronutrient and micronutrient values, their proportions, and energy contributions. In addition, provide detailed nutrition recommendations for type 2 diabetes.*

Supplementary Text S2. Full Version of Reference Diet and AI-Generated Diets

This section presents the full versions of the reference diet used in the study and the artificial intelligence-generated diets.

Important Note: Some references appearing in the AI-generated dietary suggestions in the Supplementary Materials were generated by the language models and were not selected by the authors. These references are not intended to support the scientific arguments of this study.

Reference Diet

Breakfast

- Unsweetened, light tea
- Sliced salad consisting of cucumber, tomato, and seasonal leafy greens
- 1 slice of white cheese (30 g)
- 5–6 black olives
- 2 thin slices of whole wheat bread (50 g)

Mid-Morning Snack

- 1 serving of fruit
- 2 walnuts
- 1 bowl of yoğurt (200 mL) or 200 mL milk

Lunch

- 1 bowl of soup (without cream or grain components such as barley)
- 1 serving of vegetable dish (5–6 tablespoons; with 1 tablespoons olive oil)
- 1 bowl of yogurt (200 mL)

- 2 thin slices of whole wheat bread (50 g)

Afternoon Snack

- 1 serving of fruit
- 8–10 almonds or hazelnuts (unsalted, unroasted)

Dinner

- 90 g grilled chicken
- Seasonal vegetable salad or baked vegetables prepared with 1 teaspoon of olive oil
- 200 mL ayran or yogurt
- 4–5 tablespoons of bulgur pilaf

Evening Snack

- 1 slice of white cheese (30 g)
- 1 large whole-grain cracker or 1 thin slice of whole wheat bread
- Cucumber and tomato (no added oil)

ChatGPT-5 Auto

Below is a 3-day, 1800 kcal sample diet plan for individuals with Type 2 Diabetes, prepared based on national (Republic of Türkiye Ministry of Health Nutrition Guide 2015; Türkiye Diabetes Foundation recommendations) and international (ADA – American Diabetes Association, EASD, WHO) guidelines. The plan is detailed along with carbohydrate–protein–fat ratios, micronutrients, portions, and daily recommendations.

1. General Nutrition Principles (Type 2 Diabetes)

Principle	Explanation
🍞 Carbohydrate	About 45–50% of daily energy should come from complex carbohydrates. Simple sugars should be avoided. Low glycemic index grains (whole wheat, buckwheat, oats, etc.) should be preferred.
🍗 Protein	Should be about 20% of daily energy. Lean meat, fish, eggs, legumes, yogurt, and milk are important sources.
🫒 Fat	About 30–35% of daily energy should come from healthy fats. Saturated fat <10%, and trans fat should be 0 if possible. Sources such as olive oil, avocado, and walnuts are recommended.
🧂 Salt & Sugar	Salt should be <5 g/day; refined sugar should not be used.
🥦 Micronutrients & Fiber	Daily fiber intake should be 25–35 g. Magnesium, chromium, potassium, vitamin D, and B-group vitamins should be supported.
🕓 Meal Pattern	3 main meals + 2–3 snacks are recommended. For blood glucose balance, intervals between meals should be 3–4 hours.
🚶 Lifestyle	Regular physical activity (at least 150 min/week moderate intensity), adequate sleep, and stress management are recommended.

2. 3-Day 1800 kcal Type 2 Diabetes Diet Plan

Macronutrient Distribution (Daily Average)

- Energy: 1800 kcal
- Carbohydrate: ~210 g (≈47%)
- Protein: ~90 g (≈20%)
- Fat: ~60 g (≈30%)
- Fiber: 28–32 g
- Cholesterol: <200 mg
- Sodium: <5 g salt equivalent

Day 1

Meal	Content	Kcal	CHO (g)	P (g)	F (g)
Breakfast	1 slice whole wheat bread, 1 boiled egg, 1 slice low-salt white cheese (30 g), tomato, cucumber, 5 olives, 1 tea (unsweetened)	350	25	17	18
Snack	1 apple (150 g), 10 raw almonds	150	18	3	7

Lunch	1 bowl lentil soup, 4 tbsp olive-oil vegetable dish, 1 slice whole wheat bread, 1 small bowl yogurt	450	55	20	15
Snack	1 kiwi, 1 slice whole wheat bread with labneh (10 g)	150	22	5	4
Dinner	120 g grilled chicken, 1 bowl salad + 1 tsp olive oil, 4 tbsp buckwheat pilaf, 1 bowl cacık	550	40	35	15
Night	1 glass semi-skimmed milk + 1 tsp cinnamon	150	20	10	5

Fiber: 30 g • Sodium: 4.2 g • Potassium: High • Vitamin D: 30% daily value (egg, milk, yogurt)

3. Micronutrient Values (Daily Average)

Nutrient	Amount	Daily Value Coverage
Calcium	850–1000 mg	85–100%
Magnesium	320–380 mg	100%+ (whole grains, nuts, leafy greens)
Potassium	3200–3700 mg	90–100%
Iron	12–14 mg	100%
Zinc	10–12 mg	100%
Vitamin D	6–10 µg	30–50% (sun + supplement if needed)
Fiber	28–32 g	100%+

4. Special Recommendations for Diabetes

- Prefer low glycemic index foods (buckwheat, whole grains, vegetables).
- Avoid white bread, rice, fruit juice, sugary beverages.
- Skipping meals → creates hypoglycemia risk; eating at regular times is important.
- In snacks, fiber + protein combinations reduce blood sugar fluctuations.
- A1C target is generally <7%, individualized.
- At least 1.5–2 L water per day; avoid carbonated beverages.
- Avoid frying; cooking methods should be grilling, boiling, steaming.
- 10–15 min light walks after meals reduce postprandial glucose.

ChatGPT-5 Instant

Below is a 3-day sample diet plan optimized for type 2 diabetes at an approximate daily energy level of ~1,800 kcal, prepared in light of national and international nutrition guidelines (e.g., ADA, Diabetes Canada, scientific literature reviews), together with macro/micronutrient values and recommendations. This plan represents a template; individual conditions (age, sex, kidney function, medication use, activity level, etc.) should be taken into account and the plan should be adapted with a dietitian.

Basic Principles and Guidelines in Diabetes Nutrition

Macronutrient distribution and energy ratios

• In individuals with type 2 diabetes, energy intake should be adjusted according to body weight, target HbA_{1c} level, kidney function, and medication use. In ADA and other nutrition guidelines, macronutrient ratios are generally given as follows:

- Carbohydrate: approximately 45%–60% of total energy (individualization is important)
- Protein: approximately 15%–20% of total energy
- Fat: approximately 20%–35% of total energy

• Trans fats should be avoided; saturated fat should not exceed 7%–10% of total energy, and unsaturated fats (MUFA, PUFA) should be preferred

• Dietary fiber intake is important: total daily fiber 30–50 g, especially soluble (viscous) fiber, is beneficial for vascular health and glucose control

• Added sugar (free sugar) intake should not exceed 10% of total energy (preferably lower)

• Whole grains, legumes, vegetables, fruits, and dairy products should be preferred as carbohydrate sources; refined carbohydrates (white bread, sugary foods) should be limited

• Meal timing (e.g., 3 main meals + 1–2 snacks), regular eating intervals, and distribution of carbohydrates within meals are important

• Salt (sodium) intake should be limited (e.g., <2,300 mg/day or lower, especially if hypertension is present)

• Cardiovascular risk is high; therefore, control of cholesterol, saturated fat, and trans fat is important

• If weight loss is needed, gradual loss (5%–10% of body weight) is ideal and improves glucose control in most individuals with diabetes

Based on these principles, a 1,800-kcal plan is designed. Suggested macronutrient distribution is shown below:

**Nutrient**	**Percentage (%)**	**Energy (kcal)**	**Approximate grams**
Carbohydrate	~50%	900 kcal	~900 ÷ 4 = 225 g

Protein	~18%	324 kcal	~324 ÷ 4 = 81 g
Fat	~32%	576 kcal	~576 ÷ 9 = 64 g

This distribution keeps carbohydrate intake at a "moderate" level, provides adequate protein, and keeps fat within ideal limits. (These ratios may vary according to individual needs based on the guidelines above.)

In addition, with respect to micronutrient targets (vitamins and minerals), the plan should include a variety of sources such as vegetables, fruits, whole grains, lentils, dairy products, fatty fish, and nuts in order to meet calcium, magnesium, potassium, B vitamins, vitamins A, C, E, D, iron, zinc, and other minerals.

3-Day Sample 1,800 kcal Diet Plan

Below are breakfast, lunch, dinner, and two snack suggestions for each day, together with approximate nutrient values. Food amounts are approximate and may require individual adjustment.

General Reminders

• Try to distribute carbohydrates, protein, and fat evenly at each meal; excessive carbohydrate load in a single meal may cause glucose spikes.

• Choose slowly digested (low glycemic index) carbohydrate sources (whole grains, oats, legumes, vegetables).

• Choose fat sources from unsaturated fats (olive oil, nuts, avocado, fish oil).

• Drink sufficient water daily.

• Reduce salt and limit processed foods.

• Try to consume meals at regular times.

Day 1

Breakfast (~200-220 kcal | CHO ~25–30 g • P ~14 g • F ~8–10 g)

- 1 slice whole wheat bread (~30 g)
- 2 eggs (boiled or omelet cooked with little oil)
- 1 small tomato + cucumber + green pepper
- 1 teaspoon olive oil (in salad)
- 100 g low-fat yogurt

Snack (before lunch) (~120-140 kcal | CHO ~18-20 g • P ~2 g • F ~7-8 g)

- 1 medium apple (~150 g) + 10 almonds

Lunch (~300-340 kcal | CHO ~35-40 g • P ~25 g • F ~10-12 g)

- 100 g grilled chicken breast
- 1 bowl (~200 g) vegetable dish (zucchini, pepper, eggplant, tomato, etc.)
- 1/2 cup (~50 g) bulgur pilaf

- 1 small bowl seasonal salad (lemon–olive oil dressing)

Snack (afternoon) (~60-80 kcal | CHO ~8-10 g • P ~5-6 g • F ~2-3 g)

- 1 small bowl plain low-fat yogurt + 1 teaspoon chia seeds

Dinner (~300-350 kcal | CHO ~30-35 g • P ~25-28 g • F ~15-18 g)

- 120 g grilled salmon (or fatty fish)
- 150 g steamed broccoli + carrot
- 1 small portion (~100 g) whole wheat pasta or brown rice
- 1 teaspoon olive oil (on vegetables)

Evening (optional) (~80-90 kcal | CHO ~15 g • P ~5 g • F ~3 g)

- 1 slice whole wheat bread + low-fat cheese

Estimated totals for Day 1

• Total energy ≈ 1,800 kcal

• Carbohydrate ~220–230 g → ~880–920 kcal (~49–51%)

• Protein ~75–85 g → ~300–340 kcal (~17–19%)

• Fat ~60–70 g → ~540–630 kcal (~30–35%)

Other micronutrients (fiber, vitamins, minerals) are aimed to be adequately covered through vegetables, fruits, whole grains, and fish/meat sources.

Estimated totals for Day 3

• Energy ≈ 1,800 kcal

• Carbohydrate ≈ 210–230 g (~47–51%)

• Protein ≈ 70–85 g (~16–19%)

• Fat ≈ 55–65 g (~28–33%)

Macronutrient / Micronutrient Calculation and Ratio Evaluation

Across all three days, carbohydrate, protein, and fat ratios are approximately close to the planned 50% / 18% / 32% distribution. This represents a moderate and safe distribution for individuals with diabetes (45–60% carbohydrate, 15–20% protein, 20–35% fat supported by guidelines).

From a micronutrient perspective, vitamins A, C, E, B vitamins, and minerals such as calcium, magnesium, potassium, iron, zinc, phosphorus, and iodine are aimed to be provided through vegetables, fruits, dairy products, fish, nuts, and whole grains.

Examples:

• Fiber: ~25–40 g/day (soluble fiber ~8–15 g/day)

• Calcium: >300–400 mg/day from dairy

• Omega-3 fatty acids: several servings of fatty fish per week

Not all micronutrients (e.g., vitamin B12, vitamin D, iodine, selenium) are listed in gram-level detail; however, dietary variety is intended to cover these needs.

Nutrition Recommendations and Points to Consider for Type 2 Diabetes

1. Carbohydrate control (counting/estimation/limitation)
2. Preference for low glycemic index carbohydrates
3. Increase fiber intake
4. Prefer healthy fats
5. Moderate protein intake
6. Regular meal timing and portion control
7. Adequate fluid intake and avoidance of sugary beverages
8. Sodium restriction
9. Weight management
10. Regular physical activity
11. Regular monitoring and personalization
12. Frequently monitored risks (hypoglycemia, kidney function, complications, vitamin/mineral deficiencies)
13. Non-dietary factors (sleep, stress, smoking cessation, alcohol use)

ChatGPT-5 Thinking

Great—below I provided a 3-day sample plan for type 2 diabetes at 1800 kcal, together with macronutrient ratios, approximate micronutrient targets, and practical recommendations. The plan is compatible with ADA 2025, IDF 2025, the Turkey Diabetes Guideline, and general healthy nutrition guidelines. Daily targets were set as: saturated fat <10% (preferably <6%), trans fat ≈0, added sugar <10% (preferably <5%), and sodium ≤2,000 mg.

Daily Calorie and Macro Target (same each day)

- Energy: ~1800 kcal
- Carbohydrate: 45% ≈ 200–210 g (810–840 kcal)
- Protein: 20% ≈ 90 g (360 kcal)
- Fat: 35% ≈ 70 g (630 kcal)
- Saturated fat: ≤12–18 g (AHA preference ≤6%; general limit <10%)
- Fiber: ≥30–35 g/day
- Sodium: ≤2,000 mg/day; Potassium: >3.5 g/day (if no renal restriction)
- Carbohydrate distribution (suggestion): Breakfast ~45–60 g, lunch ~45–60 g, dinner ~45–60 g, snack(s) 15–30 g. (Spreading it evenly throughout the day improves glycemia.)

3-Day Sample Menu

Note: Portions are "approximate"; there may be small deviations depending on the products you use at home.

Day 1 (≈1800 kcal)

Breakfast (~480 kcal | CHO 50 g • P 28 g • F 20 g)

- Menemen with 1 whole egg + 2 egg whites (tomato, red pepper, 1 tsp olive oil)
- 1 thin slice whole wheat bread (30 g)
- 1 slice low-fat white cheese (~40 g)
- 5–6 olives (unsalted/low-salt)
- Cucumber + greens + lemon

Snack (~180 kcal | CHO 20 g • P 8 g • F 6 g)

- Yogurt 200 g (low-fat) + 1 kiwi

Lunch (~520 kcal | CHO 55 g • P 30 g • F 18 g)

- Grilled chicken breast 120–140 g
- Bulgur pilaf ½ cup cooked (~80 g)
- Seasonal salad (1 tsp olive oil + lemon)
- Ayran 200 ml (low-salt)

Snack (~150 kcal | CHO 15 g • P 5 g • F 8 g)

- Raw almonds 15 g + 1 medium peach (or apple)

Dinner (~470 kcal | CHO 50 g • P 22 g • F 18 g)

- Olive-oil lentil soup, 1 ladle
- Olive-oil grilled vegetables (zucchini, eggplant, pepper; total 1.5 tsp oil)
- Brown bread 1–1.5 slices (40–45 g)
- Cacık (200 g yogurt + cucumber, garlic; salt restricted)

Day 1 approximate micros: Fiber 33–36 g, Sodium ~1600–1900 mg, Potassium ~4000–4500 mg, Calcium ~900–1000 mg, Magnesium ~350 mg, Iron ~12 mg, Vitamin D: limited from yogurt/cheese—if needed, evaluate vitamin D supplementation with a physician/dietitian.

Total Daily Macro Summary (each day)

CHO ~200–210 g (45%) • Protein ~90 g (20%) • Fat ~70 g (35%)

Fiber ≥30–35 g, Sodium ≤2,000 mg, Saturated fat ≤12–18 g, Trans fat ≈0, Added sugar ≤20–45 g (preferably ≤20 g). These limits are within the ranges recommended for glycemic control, cardiovascular risk, and blood pressure management.

Evidence-Based Nutrition Recommendations for Type 2 Diabetes

Personalization: Diabetes medical nutrition therapy should be individualized (culture, preferences, budget, medications, comorbidities). For example, Mediterranean, vegetarian, low-fat, or carefully monitored low-CHO patterns can be applied.

CHO quality & distribution: Spread CHO from whole grains, legumes, vegetables, and fruits evenly throughout the day; prefer high fiber and low glycemic index. Avoid sugar-sweetened beverages.

Fat quality: Emphasize mono-/polyunsaturated fats such as olive oil, hazelnuts/walnuts, and fish; keep saturated fat <10% (preferably <6%) and trans fat close to 0.

Protein: ~1.0–1.2 g/kg/day is suitable for most adults (if no kidney disease). Balance plant protein sources (legumes) with lean animal sources.

Sodium & blood pressure: Limit sodium to ~2 g/day; prefer fresh/home-prepared foods and reduce brined/processed items.

Weight management: In overweight individuals, calorie deficit and behavioral support lead to meaningful reductions in HbA1c.

Micronutrients: Vitamin D insufficiency is common; evaluate with a physician. Foods rich in magnesium, potassium, and fiber (legumes, dark leafy greens) are beneficial. Routine supplementation should be individualized.

Eating pattern: 3 main meals + 1–2 snacks; focus on consistency and not skipping meals. If you use medications with hypoglycemia risk (insulin/sulfonylureas), carry a 15 g CHO snack.

Beverages: Water, unsweetened tea/coffee (based on caffeine tolerance). Limit fruit juice in portion and frequency.

Turkish national context: The Turkey Diabetes Guideline and Turkey Diabetes Program emphasize that medical nutrition therapy should be planned individually with a dietitian and conducted together with exercise.

Frequently Asked Mini Values

- 1 g CHO = 4 kcal, 1 g protein = 4 kcal, 1 g fat = 9 kcal
- 1 slice of bread (30–35 g whole wheat) ≈ 15 g CHO
- ½ cup cooked bulgur/rice/quinoa ≈ 20–25 g CHO
- 1 cup milk/kefir (200 ml) ≈ 10 g CHO

Swap & Substitution Tips

- Instead of bulgur: buckwheat/quinoa/whole wheat pasta (similar CHO).
- Instead of yogurt/kefir: lactose-free/low-fat alternatives (similar protein).
- Instead of salmon: mackerel, sardines (similar omega-3).
- Instead of bread: whole-grain lavash/bran crackers (choose high fiber, low salt).

Important Note

This plan is a general example. Personalize it together with your dietitian based on your medical status (medications, kidney function, lipids/blood pressure, IBS/lactose intolerance, etc.). ADA/IDF/Turkish guidelines recommend individualized medical nutrition therapy and regular monitoring during follow-up (weight, waist circumference, blood pressure, A1c, lipids).

Claude Sonnet 4.5

Prepared in line with the American Diabetes Association (ADA) 2025 and international guidelines.

DAILY MACRONUTRIENT DISTRIBUTION

Total Energy: 1800 kcal/day

Macronutrient Ratios

- **Carbohydrate:** 810 kcal (202 g) – 45%
- **Protein:** 360 kcal (90 g) – 20%
- **Fat:** 630 kcal (70 g) – 35%
  - **Saturated fat:** <90 kcal (<10 g) – <5%
  - **Monounsaturated fat:** ~350 kcal (~39 g) – 19–20%
  - **Polyunsaturated fat:** ~180 kcal (~20 g) – 10%
- **Fiber:** 25–35 g/day (minimum 25 g)

🍽️ DAY 1: DETAILED MENU

BREAKFAST (400 kcal | CHO 58 g • P 16 g • F 12 g • Fb 8 g)

- Rolled oats (40 g dry), prepared with water
- Skim milk (1 glass, 200 ml)
- Strawberries (150 g, 8–10 pieces)
- Almonds (15 g, 10–12 pieces)
- Cinnamon (optional)

SNACK 1 (150 kcal | CHO 20 g • P 2 g • F 7 g • Fb 4 g)

- Apple (1 medium, 150 g)
- Walnuts (10 g, 3–4 halves)

LUNCH (500 kcal | CHO 50 g • P 40 g • F 16 g • Fb 8 g)

- Grilled chicken breast (120 g cooked)
- Bulgur pilaf (1 bowl, 150 g cooked)
- Seasonal salad (tomato, cucumber, greens – 200 g)
- Olive oil (1 tablespoon, 10 ml)
- Lemon-yogurt dressing (50 g yogurt)

SNACK 2 (150 kcal | CHO 18 g • P 12 g • F 3 g • Fb 0 g)

- Skim yogurt (150 g)

- Ginger (fresh, small amount)

DINNER (500 kcal | CHO 45 g • P 40 g • F 18 g • Fb 12 g)

- Baked salmon (100 g)
- White beans (1 bowl, 200 g)
- Boiled broccoli (150 g)
- Olive oil (1 teaspoon, 5 ml)
- Mixed green salad (100 g)

LATE EVENING (100 kcal | CHO 11 g • P 8 g • F 2 g • Fb 0 g)

- Kefir (1 glass, 200 ml)

DAY 1 TOTAL NUTRITION VALUES:

- Energy: 1800 kcal
- Carbohydrate: 202 g (45%)
- Protein: 118 g (26%)
- Fat: 58 g (29%)
- Fiber: 32 g

MICRONUTRIENT RECOMMENDATIONS (Daily Targets)

Vitamins

- Vitamin B1 (Thiamine): 1.2 mg (whole grains, legumes)
- Vitamin B6: 1.5 mg (chicken, fish, almonds)
- Vitamin B12: 2.4 mcg (meat, fish, eggs) – those using metformin should be monitored
- Folate: 400 mcg (leafy greens, legumes)
- Vitamin C: 90 mg (citrus fruits, peppers, broccoli)
- Vitamin D: 600–800 IU (fatty fish, sunlight)
- Vitamin E: 15 mg (almonds, hazelnuts, olive oil)
- Vitamin A: 900 mcg RAE (carrots, spinach)

Minerals

- Calcium: 1000–1200 mg (dairy, leafy greens)
- Magnesium: 400–420 mg (nuts, whole grains, green vegetables)
- Potassium: 2600–3400 mg (fruits, vegetables, legumes)

- Zinc: 11 mg (meat, legumes, seeds)
- Chromium: 35 mcg (broccoli, whole grains)
- Sodium: <2300 mg (salt restriction)
- Iron: 8–18 mg (red meat, legumes, spinach)

Key Omega-3 Fatty Acids

- EPA + DHA: 250–500 mg/day (fatty fish: salmon, mackerel, sardines)
- Consuming 2 servings of fatty fish per week is recommended

📋 EVIDENCE-BASED NUTRITION RECOMMENDATIONS FOR TYPE 2 DIABETES

🎯 CORE PRINCIPLES (ADA 2025 Guideline)

1. Individualized Approach

- A one-size-fits-all diet plan is not scientifically supported
- Personal preferences, cultural characteristics, and lifestyle should be considered
- Medical Nutrition Therapy (MNT) is part of the core treatment

2. Carbohydrate Management

- Reducing total carbohydrate intake improves blood sugar
- Choose high-quality carbohydrates: whole grains, legumes, vegetables, fruits
- Prefer low glycemic index (GI) foods
- Avoid added sugar and refined grains
- Drink water instead of sugary beverages

3. Fiber Intake

- Target: 30–50 g/day (minimum 25 g)
- Soluble fiber: 10–20 g/day (blood sugar and cholesterol control)
- Sources: whole grains, legumes, vegetables, fruits, oats, barley

4. Fat Selection

- Saturated fat: <9% of total energy (cardiovascular protection)
- Avoid trans fats completely
- Monounsaturated fats (MUFA): olive oil, avocado, hazelnuts
- Polyunsaturated fats (PUFA): fish oil, omega-3

- Fatty fish twice per week (150–200 g portions)

5. Protein Intake

- General recommendation: 1.0–1.2 g/kg body weight
- If diabetic kidney disease: 0.8 g/kg (caution)
- Increase plant-based protein sources (legumes, soy, nuts)
- Prefer lean meat, poultry, fish
- Limit red meat intake

🥗 DIETARY PATTERNS

Mediterranean Diet (Most Recommended)

- Plenty of vegetables, fruits, whole grains
- Olive oil as the main fat source
- Fatty fish (2–3 times/week)
- Small amounts of red meat
- Limited dairy
- Nuts and seeds

DASH Diet

- For hypertension control
- Low sodium (<2300 mg)
- High potassium, magnesium, calcium
- Plenty of fruits and vegetables

Plant-Forward Diet

- Plant-based protein sources
- Whole foods (unprocessed)
- High in fiber
- Anti-inflammatory effects

⚠️ THINGS TO AVOID

Avoid Completely

- Sugary drinks (soda, fruit juice, energy drinks)
- Products containing trans fats (processed foods, cakes, biscuits)

- Processed meats (salami, sausage, sucuk)
- Foods with added sugar
- Refined flour products (white bread, pasta)
- High glycemic index foods

Limit

- Red meat (1–2 times/month)
- Saturated fats (butter, solid margarine)
- Salt (daily <2300 mg, ideally <2000 mg)
- Alcohol (women max 1 standard drink/day, men max 2 standard drinks/day)

💡 PRACTICAL NUTRITION TIPS

Meal Timing

- 3 main meals + 2–3 snacks/day
- Do not skip meals (can cause blood sugar imbalance)
- Eat dinner early (before 19:00)
- Keep snacks at regular times

Portion Control

- Plate method: ½ vegetables, ¼ protein, ¼ whole grains
- Use smaller plates
- Eat slowly, chew thoroughly
- Stop when you feel full

Cooking Methods

- Prefer: boiling, steaming, baking, grilling
- Avoid: deep-frying
- Use minimal fat (olive oil, preferably cold-pressed)
- Use spices and herbs instead of salt

Shopping Tips

- Go to the market when you are not hungry
- Learn to read labels
- Buy fresh seasonal fruits and vegetables

- Avoid processed foods
- Stock legumes and whole grains

🔍 FACTORS AFFECTING BLOOD SUGAR

Glycemic Index (GI) and Glycemic Load (GL)

- Low GI foods (<55): legumes, oats, brown rice, most fruits
- Medium GI foods (56–69): whole-grain bread, basmati rice
- High GI foods (>70): white bread, potatoes, sugary foods

What Helps Stabilize Blood Sugar

- Fiber (slower absorption)
- Protein (satiety)
- Fat (reduces glycemic response)
- Vinegar (1–2 tablespoons before meals)
- Cinnamon (may improve insulin sensitivity)

🏃 PHYSICAL ACTIVITY

Exercise Recommendations

- Aerobic: minimum 150 minutes/week (brisk walking, swimming, cycling)
- Resistance training: 2–3 days/week (preserving muscle mass)
- Check blood sugar before/after exercise
- Hydration is important

💊 DRUG–NUTRIENT INTERACTIONS

For Those Using Metformin

- Check vitamin B12 level once per year
- Supplement B12 if needed
- Eat with or after taking the medication

For Those Using Insulin/Sulfonylureas

- Risk of hypoglycemia (low blood sugar)
- Do not skip snacks
- Be careful with alcohol
- Take precautions before exercise

📈 FOLLOW-UP AND MONITORING

Regular Checks

- Fasting blood glucose: daily (target: 80–130 mg/dL)
- Postprandial blood glucose: 2 hours after meals (target: <180 mg/dL)
- HbA1c: every 3 months (target: <7%)
- Body weight: weekly
- Blood pressure: regularly
- Lipid profile: 1–2 times/year

Targets

- Weight loss: if overweight, 5–10% (improves glycemic control)
- Waist circumference: men <94 cm, women <80 cm
- Blood pressure: <140/90 mmHg (ideal <130/80)
- LDL cholesterol: <100 mg/dL (if cardiovascular risk present, <70)

⚡ SPECIAL SITUATIONS

During Hypoglycemia

- 15 g fast-absorbing carbohydrate (3–4 sugar cubes, 150 ml fruit juice)
- Re-check after 15 minutes
- Repeat if necessary
- Follow with a protein-containing snack

During Sick Days

- Check blood sugar more often
- Increase fluid intake
- Soft, easy-to-digest foods
- Do not stop medications; consult your physician

Eating Out

- Prefer grilled, baked, boiled dishes
- Ask for sauces and sides separately
- Pay attention to portion control
- Eat salad first

SOURCES

This nutrition plan is based on the following up-to-date guidelines:

1. American Diabetes Association (ADA) Standards of Care 2025
2. Dietary and Nutritional Guidelines for People with Diabetes (2023)
3. European Association for the Study of Diabetes Nutrition Guidelines (2023)
4. Diabetes Canada Clinical Practice Guidelines
5. Endotext - Dietary Advice for Individuals with Diabetes (2024)

IMPORTANT NOTES

1. These are general recommendations. For an individualized plan, be sure to work with a dietitian or nutrition specialist.
2. If you are using medication, coordinate your nutrition plan with your physician.
3. If kidney disease is present, protein intake should be restricted (0.8 g/kg).
4. If pregnancy, breastfeeding, or other health issues are present, special planning is required.
5. Blood glucose monitoring should be done regularly and the plan should be adjusted according to results.
6. Sustainability is the most important factor. Make lifestyle changes permanent.

LONG-TERM GOALS

- Blood sugar control (HbA1c <7%)
- Healthy weight loss and maintenance
- Reducing cardiovascular risk
- Preventing/delaying diabetes complications
- Improving quality of life
- Maintaining independent living

Remember: In Type 2 diabetes management, nutrition, physical activity, medication therapy, and regular follow-up form an integrated whole. The best results are achieved through teamwork.

Gemini 2.5 Flash

Great request! Let's prepare a detailed 3-day, 1800 kcal diet plan for type 2 diabetes based on national and international nutrition guidelines (Turkish Diabetes Society, American Diabetes Association – ADA, etc.).

Important Note: This plan is a general recommendation. Nutrition therapy for individuals with type 2 diabetes should be prepared individually by a dietitian, considering age, sex, body weight, physical activity level, blood glucose control, and any other health conditions. Please consult your physician and dietitian before following this diet.

3-Day Sample 1800 kcal Diet Plan for Type 2 Diabetes

Macronutrient and Micronutrient Values and Ratios (average values based on ADA and national guidelines)

**Nutrient**	**Energy Ratio (%)**	**Calories (kcal)**	**Amount (grams)**	**Comment on Recommended Range**
Carbohydrate (CHO)	45–55%	810–990	202.5–247.5	Generally 45–55% is recommended. Complex CHO should be preferred.
Protein (P)	15–20%	270–360	67.5–90	This range is appropriate for individuals with normal kidney function.
Fat (F)	25–35%	450–630	50–70	Saturated fat should be <7%. Monounsaturated and polyunsaturated fats (olive oil, nuts) should be preferred.
Total Energy	100%	1800	—	—
Fiber	—	—	≥30	At least 30 g/day is recommended (guidelines often state 25–35 g).
Sodium (Salt)	—	—	≤2300 mg	If hypertension is present, 1500 mg should be targeted.

Sample Plan Macro Distribution (Daily Average, Approx.)

- Energy: 1800 kcal
- Carbohydrate: 225 g (≈900 kcal – 50%)
- Protein: 80 g (≈320 kcal – 18%)
- Fat: 64.4 g (≈580 kcal – 32%)
- Fiber: ≥30 g

3-Day Sample Diet List (1800 kcal)

General Principle: In each meal, including a protein, fiber, and healthy fat source alongside the carbohydrate source aims to support a more stable rise in blood glucose.

Meal	Day 1
**Breakfast (07:30)**	2 slices whole wheat bread, 60 g low-fat cheese, 1 boiled egg, plenty of raw vegetables (tomato, cucumber, pepper, greens)
**Snack 1 (10:00)**	1 portion small fruit (e.g., 1 small apple) + 10 raw almonds
**Lunch (12:30)**	1 portion (8 tbsp) olive-oil green beans (low oil), 4 tbsp homemade yogurt (semi-skim), 1 slice whole wheat bread
**Snack 2 (15:30)**	1 glass kefir + a palm-sized portion of walnuts
**Dinner (18:30)**	120 g grilled/baked fish (e.g., salmon/sea bream), large bowl steamed vegetables (broccoli, cauliflower), green salad (no oil)
**Snack 3 (21:00)**	1 glass semi-skim milk + cinnamon

Additional notes:

- Cooking methods: Prefer grilling, baking, steaming, or boiling instead of frying.
- Oil use: Add a total of 2–3 teaspoons of olive oil per day across meals and salads.
- Beverages: Unsweetened tea/coffee, plenty of water (8–10 glasses/day).
- Salt: Limit salt intake (<5 g/day).

Nutrition Recommendations for Type 2 Diabetes (National and International Guidelines)

1) Carbohydrate Management

- **Quality first:** Carbohydrates should make up 45–55% of energy. Prefer complex sources (whole grains, legumes, vegetables); strictly limit simple sugars (table sugar, honey, jam, syrupy desserts, soft drinks, fruit juices).
- **Fiber intake:** Target ≥30 g/day. Fiber slows glucose rise and increases satiety. Get it from whole grains (whole wheat, oats, rye, bulgur, quinoa), vegetables, fruits, and legumes.
- **Glycemic index (GI) and load (GL):** Prefer low or moderate GI foods (e.g., bulgur/whole-wheat pasta instead of white rice; whole-grain bread instead of white bread).
- **Portion control:** Control CHO portions at main meals and aim for similar CHO amounts in each main meal (e.g., 45–60 g CHO per main meal).

2) Fat Management

- **Amount and quality:** Total fat should not exceed 25–35% of energy.
- **Saturated and trans fat:** Saturated fat should be <7% and trans fats should be minimized as close to zero as possible (fatty red meats, full-fat dairy, butter, margarine, offal, fried foods, processed foods).
- **Healthy fats:** Prefer MUFA (olive oil, avocado, hazelnuts, almonds) and PUFA (omega-3: fish, flaxseed, walnuts). Fish 2–3 times/week is recommended.

3) Protein Management

- **Amount:** Protein should be 15–20% of energy (≈0.8–1.2 g/kg body weight).
- **Sources:** Prefer lean meat, skinless chicken/turkey, fish, legumes, low-fat dairy (yogurt, cheese, kefir).
- **Kidneys:** If diabetic nephropathy is present, protein intake should be further restricted under physician/dietitian supervision.

4) Meal Pattern

- **Small and frequent:** A pattern of 3 main meals + 2–3 snacks is recommended. Avoid long fasting periods and keep meals at regular times. This helps prevent sharp drops and spikes in blood glucose.
- **Meal composition:** Each main meal should include complex carbohydrates + lean protein + fiber (vegetables/salad).

5) Other Key Points

- **Water intake:** At least 8–10 glasses/day.
- **Salt intake:** Limit sodium to 2300 mg/day (~1 tsp salt). Avoid processed foods, pickles/brined products, and instant soups.
- **Alcohol:** Limit or preferably avoid. If used, be very cautious and never consume on an empty stomach.
- **Physical activity:** Regular activity (at least 150 minutes/week moderate-intensity aerobic exercise) is an essential part of nutrition therapy.

These recommendations aim to support glycemic control, reduce cardiovascular risk factors, and maintain a healthy body weight in the management of type 2 diabetes.

Gemini 2.5 Pro

Important Note: This diet plan is a general example. Each individual's nutritional needs differ depending on age, weight, height, physical activity level, medications used, and blood glucose patterns. Therefore, it is vitally important to consult both a physician and a nutrition specialist (dietitian) before implementing this plan.

General Nutrition Recommendations for Type 2 Diabetes

To understand the rationale behind this diet plan, the main principles are as follows:

1. Carbohydrate Quality and Amount:

- **Choose Complex Carbohydrates:** Prefer high-fiber whole grains such as whole wheat bread, bulgur, buckwheat, and oats instead of refined carbohydrates like white bread, rice, and pasta.
- **Portion Control:** Carbohydrates directly affect blood sugar. It is important to know how much carbohydrate you consume at each meal. Your dietitian will determine suitable portions for you.
- **Avoid Sugar:** Foods containing simple sugars such as sugary beverages, desserts, pastries, and baked goods raise blood sugar very quickly. Avoid these.

2. Consume Healthy Fats:

- **Focus on Unsaturated Fats:** Olive oil, avocado, raw nuts such as hazelnuts, almonds, walnuts, and fatty fish (salmon, mackerel) contain unsaturated fats that are beneficial for heart health.
- **Limit Saturated and Trans Fats:** Avoid fatty cuts of red meat, butter, clotted cream, processed meats (salami, sausage), and trans fats found in packaged products.

3. Get Enough High-Quality Protein:

- **Lean Protein Sources:** Lean red meat, skinless chicken and turkey, fish, eggs, yogurt, legumes (lentils, chickpeas), and tofu increase satiety and help blood sugar rise more slowly.

4. Increase Fiber Intake:

- Fiber slows digestion and helps sugar enter the bloodstream more gradually. Vegetables, fruits (with portion control), whole grains, and legumes are excellent sources of fiber. The target should be at least 25–30 grams of fiber per day.

5. Portion Control and the "Healthy Plate Model":

- Fill half of your plate with non-starchy vegetables (salad, broccoli, spinach, tomatoes, etc.).
- Allocate one-quarter of your plate to lean protein sources.

- Allocate the remaining quarter to complex carbohydrates (whole grains, legumes).

6. Fluid Intake:

- Drink at least 2–2.5 liters of water per day. Strictly avoid sugary and carbonated beverages and ready-made fruit juices. Ayran (unsalted), mineral water, and unsweetened herbal teas are good alternatives.

7. Reduce Salt Intake:

- Diabetes increases the risk of high blood pressure. Avoid adding extra salt to meals and avoid very salty foods such as brined products and pickles.

Sample 3-Day 1800 kcal Type 2 Diabetes Diet Plan

Portions in this plan are average values and should be adjusted individually.

DAY 1

Breakfast (08:00) – ~450 kcal

- 1 boiled egg
- 1 slice (30 g) low-fat white cheese
- 5–6 olives (unsalted)
- Plenty of tomatoes, cucumbers, greens
- 2 slices whole wheat bread (50 g)
- Unsweetened tea

Snack (10:30) – ~150 kcal

- 1 small green apple
- 10 raw almonds

Lunch (13:00) – ~500 kcal

- 120 g grilled chicken breast (about the size of 4 meatballs)
- 4 tablespoons bulgur pilaf
- A large bowl of seasonal salad with plenty of greens, pomegranate molasses, and 1 teaspoon olive oil
- 1 glass mint, unsalted ayran

Snack (16:00) – ~150 kcal

- 1 bowl (150 g) plain yogurt (homemade or probiotic)
- 1 tablespoon rolled oats

Dinner (19:00) – ~550 kcal

- 1 serving olive-oil artichoke or leek dish (about 8 tablespoons)
- 1 bowl (200 g) lentil soup
- 1 slice whole wheat bread (25 g)
- 1 bowl cacık (with cucumber and mint)
